# Heterophil/Lymphocyte Ratio Level Modulates *Salmonella* Resistance, Cecal Microbiota Composition and Functional Capacity in Infected Chicken

**DOI:** 10.3389/fimmu.2022.816689

**Published:** 2022-04-14

**Authors:** Mamadou Thiam, Qiao Wang, Astrid Lissette Barreto Sánchez, Jin Zhang, Jiqiang Ding, Hailong Wang, Qi Zhang, Na Zhang, Jie Wang, Qinghe Li, Jie Wen, Guiping Zhao

**Affiliations:** State Key Laboratory of Animal Nutrition, Institute of Animal Sciences, Chinese Academy of Agricultural Sciences, Beijing, China

**Keywords:** H/L, diseases resistance, cecal microbiota, metagenome sequencing, *Salmonella*

## Abstract

The gastrointestinal microbiota plays a vital role in ensuring the maintenance of host health through interactions with the immune system. The Heterophil/Lymphocyte (H/L) ratio reflects poultry’s robustness and immune system status. Chickens with low H/L ratio are superior to the chickens with high H/L ratio in survival, immune response, and resistance to *Salmonella* infection, but the underlying mechanisms remain unclear. This study aimed to identify microorganisms associated with resistance to *Salmonella* Enteritidis infection in chickens based on the H/L ratio. The 16S rRNA and metagenomic analysis were conducted to examine microbiome and functional capacity between the 2 groups, and Short Chain Fatty Acids (SCFAs) and histopathology were conducted to explore the potential difference between susceptible and resistant groups at 7 and 21 days post-infection (dpi). The microbiome exploration revealed that low H/L ratio chickens, compared to high H/L ratio chickens, displayed a significantly higher abundance of *Proteobacteria* (*Escherichia coli*) and *Bacteroidetes* (*Bacteroides plebeius*) at 7 and 21 dpi, respectively. *Anaerostipes* (r = 0.63) and *Lachnoclostridium* (r = 0.63) were identified as bacterial genus significantly correlated with H/L (P < 0.001). Interestingly, *Bacteroides* was significantly and positively correlated with bodyweight post-infection (r = 0.72), propionate (r = 0.78) and valerate (r = 0.82) contents, while *Salmonella* was significantly and negatively correlated with bodyweight post-infection (r = − 0.67), propionate (r = − 0.61) and valerate (r = − 0.65) contents (P < 0.001). Furthermore, the comparative analysis of the functional capacity of cecal microbiota of the chickens with high and low H/L ratio revealed that the chickens with low H/L ratio possess more enriched immune pathways, lower antibiotic resistance genes and virulence factors compared to the chickens with high H/L ratio. These results suggest that the chickens with low H/L ratio are more resistant to *Salmonella* Enteritidis, and it is possible that the commensal *Proteobacteria* and *Bacteroidetes* are involved in this resistance against *Salmonella* infection. These findings provide valuable resources for selecting and breeding disease-resistant chickens.

## Introduction

The world population is constantly increasing and is expected to reach approximately 9.6 billion people by 2050. Equivalently, poultry production has accelerated in recent years, with an estimated 130 million tons of chicken meat produced in 2020 (1) to meet the demands of a growing global population. Between 2020 and 2029, the Organization for Economic Cooperation and Development (OECD) and the United Nations Food and Agriculture Organization (FAO) forecast that poultry farming will increase by 1.5 percent. Such significant growth is only possible with effective disease control and prevention strategies that minimize the impact of bacterial, parasitic, or viral infections on animals while also minimizing associated ecological damage and resource waste (2). Unfortunately, *Salmonella* infections threaten the poultry industry and public health. Although spontaneous *Salmonella* spp. infection will not result in a high number of chicken deaths, it will have a significant negative effect on poultry production capacity and health. Furthermore, it is a zoonosis, posing a major threat to public health and safety (3–7). Therefore, it is crucial to understand the host immune response and mechanisms of resistance to *Salmonella* diseases to minimize economic losses in poultry production and protect animal and human health (8).

Chickens’ gut microbiota is diverse and complex, and it is crucial not only for nutrition but also for immune system development and pathogen exclusion. By adhering to the intestinal epithelial walls, the gut microbiota can form a shielding barrier, minimizing the opportunity for pathogenic bacteria to colonize (9). These bacteria can produce short chain fatty acids (SCFAs: acetate, propionate, and butyrate), organic acids (lactic acid), and antimicrobial compounds (bacteriocins), as well as eliciting non-pathogenic immune responses that provide nutrition and protection to the animal (9–11). The primary benefits of commensal bacteria include nutrition support for the host, the competitive exclusion of pathogens or non-native microbes, and immune stimulation and programming (12).

Additionally, commensal microbiota can promote the development of the immune system, which includes the intestinal epithelial cells monolayer, mucus layers, intestinal immune cells (cytotoxic and helper T cells, immunoglobulin producing cells, and phagocytic cells), and lamina propria (4, 12, 13). These tissues act as a barrier between the host and the microbes, defending the host against undesirable gut microorganisms.

By affecting host cells’ physiology and gene expression, microbial metabolites can also modulate the immune system (14–16). The SCFAs possess bacteriostatic properties that inhibit the growth of foodborne pathogens such as *Salmonella* spp. (17). Thus, it has become increasingly clear that the microbiota and its metabolites play a critical role in orchestrating host physiology and pathophysiology by regulating a wide variety of metabolic, inflammatory, and even behavioral processes (13, 18, 19).

The H/L ratio reflects the capacity to cope with infection through injury (via heterophils cells) and transmissible disease (via lymphocytes cells) (20). Heterophils form the first line of immune defense against bacterial pathogens in inflammatory lesions. In contrast, lymphocytes play a central role in humoral adaptative immunity (B cells) and cell-mediated adaptative immunity (T cells) (21). The H/L ratio is generally described as a robust measure of physiological stress in birds. Nevertheless, a piece of empirical evidence demonstrated that the H/L ratios are positively correlated with the strength of the innate immune response (22). Chickens with low H/L are superior to the chickens with high H/L in survival, immune response, and resistance to *Salmonella* infection (21, 23–26). To our knowledge, no study emphasized the effects of different H/L ratio levels on the microbiota composition and its outcome in chicken disease resistance.

Interestingly, low H/L ratios were identified as an ancestral state in birds, which may provide a long life span and survival (21). However, no study has been conducted to assess the effect of high and low H/L ratios on the immunomodulatory mechanisms of intestinal microbiota against *Salmonella* infection in chicken broilers. Therefore, to overcome this gap, the current study was initiated to compare the disease resistance of the high and low H/L ratio chickens infected with *Salmonella enterica* subsp. *enterica* serotype Enteritidis and establish the relationship between resisting *Salmonella* infection and the gut microbiota composition. Through this, we have identified the bacterial taxa likely to be involved in the resistance to *Salmonella* in chicken broilers. Specifically, we first examined the effect of different H/L ratio levels on the resistance and inflammatory response to S. Enteritidis (SE) infection (at 7 and 21 dpi). Then, we evaluated the intestinal barrier immune function, the SCFAs contents, and the cecal microbiome composition (at 7 and 21 dpi) of SE-infected high and low H/L ratio chickens, using 16S rRNA gene sequencing and metagenome sequencing. This may be potentially helpful for breeding disease-resistant chickens and developing a specific target to improve the chicken’s gastrointestinal tract homeostasis.

## Materials and Methods

The protocol of this study was reviewed and approved by the Institute of Animal Sciences’ Animal Welfare Committee (Chinese Academy of Agricultural Sciences, Beijing, China). Furthermore, animal experimentation and survival were approved ethically by the IAS-CAAS Animal Ethics Committee (approval number: IAS2021-31).

### Animal and Experimental Design

The chickens used in this study were obtained from a family of chickens formed by individuals with low H/L ratio, selected based on their H/L ratio at 56 days old. The 13^th^ generation of the highly inbreed genetic H/L line, Jinxing Yellow chickens from the Institute of Animal Sciences, Chinese Academy of Agricultural Science (Beijing, China), hatched at Changping experimental farm (Beijing, China), were utilized in the current study. Immediately after hatch, 360 chicks were transferred to housing rooms equipped with sterilized isolation ventilated cages (IVC) (IPQ-type 3 negative pressure isolator). The birds were randomly divided into two groups: non-infected and SE-infected. After *Salmonella* infection, the birds of each group were assigned to one IVC with an average of 100 and 200 birds, respectively, for non-infected and SE-infected groups (**Supplementary Figure 1**). Two days before the assignment of birds to each treatment, the temperature in the IVC was maintained at 37°C. Then, it was fixed at 35°C with a weekly decrease of 2°C until the experiment ended (21 days post-infection). The chicks received ad libitum Specific Pathogen Free (SPF) feed (Beijing Keao Xieli Feed Co., Ltd., Beijing, China) and open access to sterilized water throughout the experiment. Before infection, all the chicks were checked for *Salmonella* presence by culturing cloacal swab samples in buffered peptone water overnight at 37°C with agitation. After culturing overnight, the samples were spread on brilliant green agar, then incubated at 37°C (18-24 hours) (27). According to the results, no infected chicks were detected. The experimental design reported herein should be considered in the light of some limitations such as the absence of sampling before infection and non-infected group at 21 dpi, and the sample size.

### White Blood Cells Count and Determination of H/L Ratio

At 7 days old, 10 µl of fresh blood samples were collected from each bird (from the wings) and smeared on microscopic glass slides. The resulting blood smears were air-dried then stained using Giemsa staining. One hundred leukocytes were counted, including heterophils, lymphocytes, and monocytes, following a schematic diagram and using a Leica DM500 microscope with a magnification of 100x immersion oil (28). The H/L ratio was calculated by dividing the number of heterophil cells by lymphocyte cells. Differentiation of individuals into low and high H/L ratio was based on a fixed H/L ratio value (0.2) as follows: low H/L ratio = chicken with a H/L ratio < 0.2; high H/L ratio = chicken with a H/L ratio > 0.2.

### Bacterial Strain and Infection

*Salmonella* Enteritidis 50335 (Institute of Veterinary drugs Control, Beijing, China) was used to challenge the birds in this experiment. To perform the infection assay, 100 µl from a cryopreservation bank was grown at 37°C in Luria Bertani Broth (LB) with agitation (150 rpm) overnight. After concentration by centrifugation, the bacteria concentrates were resuspended in sterile phosphate-buffered saline (PBS). The final number of colony-forming units (CFUs) was determined by plating in triplicate ten times serial dilutions on Brilliant Green agar (37°C, overnight). Based on previous studies (29, 30) and according to the aim of the infection trial, which was to induce sufficient immune response of the host and ensure the detection of *Salmonella* in the immune organs, the birds from the SE-infected group were infected with 1x10^10^ CFUs of SE/ml of PBS at 7 days old. This dosage has been carefully defined based on pre-experiments, in order to fully show phenotypic differences between high and low H/L ratio chickens under *Salmonella* infection. The birds from the non-infected group received the same volume of sterile PBS.

### Samples and Phenotypical Data Collection

The samples were collected at 7 and 21 days post-infection. At each time point post-infection, 30 chickens from each experimental group (non-infected and SE-infected) were randomly selected, weighed, blood samples (1.5 ml distributed in one blood vial EDTA tube and microcentrifuge tube for serum collection) were collected from the wings and stored at − 20°C (and at room temperature for serum collection) for future analysis, and then they were slaughtered. Next, the slaughtered chickens were aseptically eviscerated. The liver and different gastrointestinal tract tissues (ileum and one ceca (section performed 2 cm from the junction ileocecal) were aseptically sampled, washed with PBS and stored in cryovial tubes at − 80°C for later analysis. In addition, sections from ileum and caecum gut segments previously sampled and washed with PBS were collected and stored in 4% paraformaldehyde for later histology analysis. After sectioning the second ceca, sterile tweezers were used to squeeze the contents into sterile cryovial tubes for SCFAs and DNA extraction for 16S and metagenome sequencing analysis. After random sampling of the birds from non-infected and SE-infected at 7 and 21 dpi, the birds from each group (non-infected and SE-infected) were divided into low and high H/L ratio chicken subgroups. We performed all the following analyses presented in this study from the subdivision of SE-infected into low and high H/L ratios.

### The Measure of IL-1β, IL-8, and IFN-γ Blood Serum Concentration

In this study, the IL-1β, IL-8, and IFN-γ concentrations in the serum were measured using Enzyme-Linked Immunosorbent Assay (ELISA) kits, according to the manufacturer’s instructions (Cusabio Biotech Co., Ltd., Wuhan, China). The assay was performed in triplicate and included three to four SE-infected chickens from the low and high H/L ratio groups at 7 and 21 dpi. In brief, serum was diluted (100-fold) for generation of a standard curve with Horseradish Peroxidase (HRP) conjugated antibody for the targeted immune factor was added to a plate precoated with the target. After incubation and wash, the intensity of the color generated due to the addition of a substrate solution was then measured by a microplate reader. Based on the standard curves, the concentration of IL-1β, IL-8, and IFN-γ in the serum was calculated.

### Nucleic Acids Extraction for *Salmonella* Load Determination

The genomic DNA (gDNA) utilized in the current study was purified using the Phenol-Chloroform method with modifications. Blood (20 µl) and cells suspended from homogenized tissues of liver, ileum, and caecum (30mg) were mixed with 800 µl of Lysis buffer (Tris 6.43%, EDTA 4.94%, NaCl 62.07%, SDS 26.56%), 4 µl of RNase A (Tiangen, Beijing, China) and 25 µl of proteinase k. The mixture was shaken vigorously by inversion for 10 minutes, then incubated at 56°C overnight. The DNA extraction procedure was carried out by adding an equal volume of phenol, chloroform, and absolute ethanol and gently shaking the solution until it became milky. After centrifuging the mixture at 12 000 rpm for ten minutes, the organic phase was removed. 1000 µl of absolute ethanol was used to precipitate the gDNA. The DNA pellet was washed with 300 µl of 75% alcohol (ethanol). At the last step, the tubes were centrifuged 8500 rpm 5min; then, the supernatant was removed, and the pellet was air-dried for 5 min at room temperature. The pellet of DNA was resuspended in 200 µl of nuclease-free water (double distilled water). The primers and probes (Sdf1) utilized in this study are listed in **Supplementary Table 1**.

### Quantitative Real-Time PCR (RT-QPCR)

To determine the bacterial (SE) load in the blood, liver, ileum, and caecum, gDNA of 3 to 6 birds from each group were used. Quantitative real-time PCR (Q-PCR) was performed using TaqMan probes and KAPA PROBE FAST qPCR Master Mix (2x) Kit (KAPA BIOSYSTEMS, US Wilmington, Massachusetts), accordioning to the manufacturer’s instructions for volume total of 20 µl of the reaction mixture. Primers and fluorescent probe (5’FAM-TAMRA3’) for *Salmonella* differentiating fragment 1 (Sdf1) (No.AF370707.1) were used in this study. To quantify total *Salmonella* load in different organs, we used a standard curve for sdf1 (y = -3.3517x + 45.296) generated from serial dilutions of constructed and transformed Sdf1 plasmids, with a starting copy number of 3.79 x 10^10^ gDets (copies) calculated using the following equation (31, 32):


$$gDets\left(copies\right)=\frac{6.02\times 10^{23}(copy/mol)\times DNAamount\left(g\right)}{DNAlength\left(dp\right)\times 660\left(g/mol/dp\right)}$$

The amplification system was as follows: 95°C for 30 s, 40 cycles of 95°C 5 s, and 60°C for 34 s, with an additional step of 60°C for 15 s at the end. The primers and the probe used in this study are listed in the **Supplementary Table 1**.

### Ileum and Caecum Goblet Cells Count, and Ileal Villi Morphological Analysis

To perform intestinal barrier immunity analysis at 7 and 21 dpi, 2 to 3 birds from high and low H/L ratio SE-infected chickens were used to analyze the goblet cells density (33) and the ileal villi morphometry (34). Ileum and caecum gut segments (2 cm long) were cut and flushed with PBS three times to remove the intestinal contents. After the abduction of intestinal contents, the tissues of each gut segment, time point post-infection, and bird were fixed in 2 ml sterile tubes with 4% paraformaldehyde for later histological examination. Paraffin blocks of 3 mm of thickness were used to produce sections of 4 µm of thickness. The latest section was fixed on a microscopic glass slide and stained using Hematotoxin and Eosin (HE) and Alcian Blue-Passive Acidification Shift (AB-PAS, for goblet cells detection). For the determination of goblet cells number (per villus or fold, and crypt), 10 villi (ileum), 5 folds (caecum), and 20 crypts representative and intact were used to count the number of goblet cells in each indicated intestinal structure. Concerning the morphometry analysis of the ileal villi, ten representative intact villi and their associated crypt were selected to measure the villus height (VH), crypt depth (CD), villus height/crypt depth ratio (VH/CD), villus width (VW), villus surface area (VSA= VH x VW x π), epithelium thickness (ET) and lamina propria thickness (LPT) using Image J (Wayne Rasband and contributors, National Institutes of Health USA). The pictures were captured using a light microscope Leica DMI6000B (Wetzlar, Germany) equipped with Leica Application Suite (LAS) image-processing software.

### SCFAs Cecal Content Concentration Analysis

This study measured the cecal contents SCFAs concentrations by Gas Chromatography-Mass Spectrometry (GC-MS). In brief, 100 mg of accurately weighted cecal contents were homogenized in a 2 ml grinding tube containing one bead and 1 ml of distilled water (containing 0.5% of phosphoric acid and 50 µg/ml of internal standard 2-ethylbutyric acid). The mixture was homogenized twice for 3 min at 50 HZ in a cryo-grinding machine and then centrifuged at 4°C, 13000 g for 15 min. Then, the supernatant was collected and transferred into a new 1.5 ml microcentrifuge tube containing 500 µl of ethyl acetate for extraction. The resulting mixture was homogenized by vortex, sonicate in an ice-water bath for 10 min, and centrifuged at 4°C, 13000 g for 10 min. Finally, the latest supernatant obtained was collected and used for GC-MS analysis using an HP-FFAP capillary column (30 m x 0.25 mm x 0.25 µm, Agilent J&W Scientific, Folsom, CA, USA) and a mass-selective flame ionization detector (FID) with the following parameters: injection volume 1 µl; split injection; split ratio 10:1; solvent delay 2.5 min. The temperature program was: the initial temperature of the column oven is 80°C, the temperature is increased to 120°C (at 40°C/min), and the temperature is increased to 200°C (at 10°C/min), and then run at 230°C for 3 min. The Chromatographic conditions were: electron bombardment ion source (EI), ion source temperature 230°C, quadrupole temperature 150°C, transmission line temperature 230°C, and electron energy 70 eV. The peaks of individual SCFAs and BSCFAs in each cecal sample were acquired following the peaks obtained using a standard solution (Sigma-Aldrich, MO, United States). In brief, the standard curve of each targeted SCFA was obtained by dividing the peak area of the target SCFA by the peak area of the standard solution. The ratio of the target’s peak area and the standard solution’s peak area was used as the ordinate and the concentration as the abscissa for drawing a linear regression line. The molar concentration of each SCFA was calculated using the ratio of the peak area of the individual SCFA and the peak area of the standard solution multiplied by the concentration of the standard solution (35). The data analysis was performed using Masshunter quantitative software Version 10.0.707.0 (Agilent, USA) with default parameters.

### Bacterial Community Profiling: DNA Extraction and 16S rRNA Gene Sequencing

The cecal microbiota richness and diversity were determined by 16S rRNA gene sequencing analysis as described previously (36), with some modifications. In brief, bacterial genomic DNA was extracted from 37 cecal content using E.Z.N.A.^®^ stool DNA Kit (Omega Bio-Tek, Norcross, GA, United States) according to the manufacturer’s instructions. The concentration and purity of the DNA extracted were determined with a NanoDrop 2000 UV-vis spectrophotometer (Thermo Scientific, Wilmington, USA). DNA quality was inspected by electrophoresis in 1% agarose gel. The hypervariable region V3-V4 of the bacterial 16S rRNA gene was amplified with the primers 338F (5’-ACTCCTACGGGAGGCAGCAG-3’) and 806R (5’-GGACTACHVGGGTWTCTAAT-3’) using an ABI GeneAmp^®^ 9700 PCR thermocycler (ABI, CA, USA). PCR reactions were performed in triplicates, and the resulting products were extracted from a 2% agarose gel. Purification and quantification were performed using the AxyPrep DNA Gel Extraction Kit (Axygen Biosciences, Union City, CA, USA) and the QuantusTM Fluorometer, respectively (Promega, USA). Equimolar amounts of purified amplicons were pooled and subjected to pair-end sequencing on an Illumina MiSeq platform (Illumina, San Diego, USA) according to the standard protocols of Majorbio Bio-Pharm Technology Co. Ltd. (Shanghai, China).

### Processing and Diversity Analysis

The Raw fastq files were demultiplexed, quality filtered by fastp version 0.20.0 (37), and merged by FLASH version 1.2.11 (38) with the following criteria (37, 38): (1) 300 bp reads were truncated at sites with an average quality score less than 20 over a 50 bp sliding window, and reads shorter than 50 bp were discarded; reads containing ambiguous characters were also discarded; (2) only overlapping sequences with a length greater than 10 bp were assembled according to their overlapped sequence. The overlap region’s maximum mismatch ratio was 0.2; (3) Samples were identified using the barcode and primers. The sequence direction was adjusted, primers were precisely matched, two nucleotide mismatches were permitted, and reads with ambiguous characters were discarded. Additionally, reads that could not be assembled were discarded. The Quantitative Insights into Microbial Ecology 2 (QIIME2) software (v2020.2) was used to analyze the microbiome diversity (39). Demultiplexed raw sequence data were quality-filtered and denoised using DADA2 method of QIIME2 (40). The deduplicated sequences were amplicon sequence variants (ASVs) with approximately 100% of identity, and the UPARSE-OUT algorithm in VSERACH software (v2.13.4_linux_x86_64) was utilized to select paired sequences at a 97% identity match to operational taxonomic units (OTUs). Next, the QIIME2 software was used to allocate the representative sequences taxonomically by searching against the SILVA (v138) 16S rRNA database using a confidence threshold equal to 0.7. The pretrained naive Bayes classifier was used for species annotation. The alpha (Shannon, Simpson, ACE, and Chao1 indices) and beta diversity metrics (weighted UniFrac PCoA plots) were used to analyze the overall cecal microbiota profile using the normalized data from each sample. Alpha diversity metrics between low and high H/L ratio chickens were evaluated with Mann-Whitney U test, while ANOSIM and ADONIS with 999 permutations was performed with reported R (R^2^ for ADONIS) and p-value for beta diversity with weighted UniFrac PCoA plots. In addition, PERMANOVA for the six groups (low and high H/L ratio control and SE-infected chickens at each dpi) were also analyzed with grouping factor, the samples grouping and the H/L ratio. Analysis of the relative abundance of the microbial community at phylum and genus levels were also assessed and visualized with bar plot, with a comparative analysis performed using Wilcoxon rank-sum test.

### Metagenomic Profiling: DNA Extraction, Library Construction, and Metagenome Sequencing

Metagenome sequencing was used to investigate the cecal microbiota in greater detail, as previously described (41), with some modifications. To summarize, total genomic DNA was extracted from the cecal contents 26 of chickens SE-infected using the E.Z.N.A.^®^ Soil DNA Kit (Omega Bio-Tek, Norcross, GA, United States) according to the manufacturer’s protocol. TBS-380 and NanoDrop2000 were used to determine the concentration and purity of extracted DNA, respectively. On a 1% agarose gel, the DNA quality was determined. The extracted DNA was then fragmented to an average size of approximately 400 bp using the Covaris M220 (Gene Company Limited, China) to construct paired-end libraries. Using NEXTflexTM Rapid DNA-Seq, a paired-end library was built (Bioo Scientific, Austin, TX, USA). To the blunt end of the fragments, adapters containing the complete complement of sequencing primer hybridization sites were ligated. On the Illumina NovaSeq/Hiseq Xten, paired-end sequencing was performed (Illumina Inc., San Diego, CA, USA) at Majorbio Bio-Pharm Technology Co., Ltd. (Shanghai, China) using NovaSeq Reagent Kits/HiSeq X Reagent Kits according to the manufacturer’s instructions (www.illumina.com).

### Sequence Quality Control and Genome Assembly

Clean reads were generated from raw reads from metagenome sequencing (42) by removing adaptor sequences, trimming, and low-quality reads (length threshold 50 bp, quality value 20, or containing N bases) using fastp version 0.20.0. (36). Following that, we aligned clean reads to the *Gallus gallus* genome (version 5.0) (43) using BWA (version 0.7.9a) to identify and remove host-originated reads. The high-quality reads were then assembled to contigs using MEGAHIT (version 1.1.2), which use succinct de-Bruijn-graphs. The quality and quantity of the scaffolds generated were evaluated, and the best (kmer_min = 47, kmer_max = 97) were selected. Contigs with lengths equal to or superior to 300 bp were chosen as the final assembling result for gene prediction and annotation.

### Gene Prediction, Taxonomy, and Functional Annotation

Metagene was used to identify the open reading frames (ORFs) in each metagenome sample (44). We chose predicted ORFs with at least 100 bp length and translated them into amino acid sequences using the NCBI translation table7. A non-redundant gene catalogue was constructed using the parameters of 90% sequence identity and 90% coverage (version 4.6.1) (42). After quality control, reads were mapped to the non-redundant gene catalogue with 95% identity using SOAP aligner (version 2.21) (43), and gene abundance from each sample was evaluated. To perform the taxonomic annotation, non-redundant gene catalogues were aligned against NCBI non-redundant database using blastp as implemented in DIAMOND (version 0.9.19) with an e-value cutoff of 1e−5 (44). The Kyoto Encyclopedia of Genes and Genomes (KEGG) annotation was conducted using Diamond against the KEGG database (version 94.2) with an e-value cutoff of 1e−5. Antibiotic resistance genes and virulence factor annotation were performed using Diamond against CARD database (version 3.0.9) and VFDB database, respectively, with an e-value cutoff of 1e−5.

### Statistical Analysis

The results are expressed as the mean and standard error of the mean. GraphPad Prism version 8 (GraphPad Software, San Diego, CA, USA) and R version 3.3.1 were used to analyze the data. The differences in SE load and inflammatory cytokine concentrations in serum were analyzed using a two-way ANOVA with Sidak’s multiple comparisons. Briefly, relative abundance differences between two groups were analyzed using the Wilcoxon rank-sum test, also known as the Mann–Whitney U test (a nonparametric test for two independent sets of samples), for comparison between low and high H/L chickens at 7 and 21 dpi, and statistical significance was declared when the p-value was < 0.05. The Kruskal-Wallis’s sum rank test was used to analyze the four groups and detect significant differential abundance features at a taxonomic level of interest (phylum and genus). Correlogram were performed using Pearson’s (intestinal histopathology) and Spearman’s correlation (association microbiota with environmental factors) using R software. Correlation between 2 factors were considered significant when the coefficient of correlation (R) was > 0.55 and p-value < 0.05.

## Results

### Low H/L Ratio Confers Resistance Advantages During S. Enteritidis Infection

**Figure 1:**
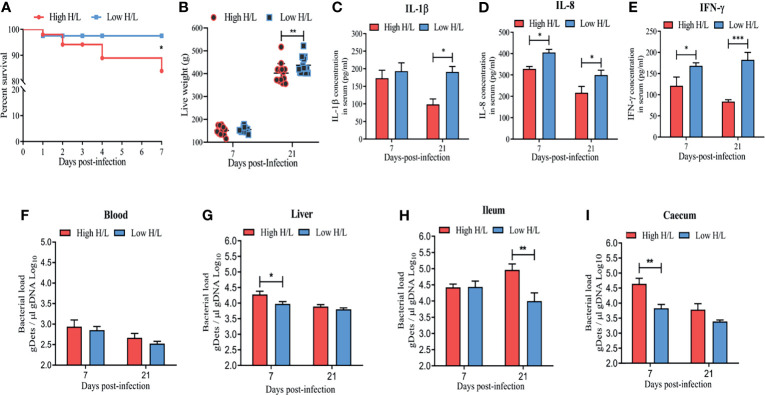
Low H/L ratio chickens exhibit resistance advantages and reduced *S*. Enteritidis load. After infection by oral gavage with 1ml of PBS containing 10^10^ CFU/ml of SE, the **(A)** Survival curve Kaplan-Meier; **(B)** body weight post-SE-infection (11-15 birds were used); **(C)** IL-1β blood serum concentration difference between low and high H/L ratio SE-infected chickens at 7 and 21 dpi; **(D)** IL-8 blood serum concentration difference between low and high H/L ratio SE-infected chickens at 7 and 21 dpi; **(E)** IFN-γ blood serum concentration difference between low and high H/L ratio SE-infected chickens at 7 and 21 dpi; Quantification of total *S*. Enteritidis gDets in different segments of the gastrointestinal tract, blood, and Liver was performed by quantifying the Sdf1 (total bacteria, *Salmonella* different fragment 1 sequence by qPCR. gDNA from Blood **(F)**, Liver **(G)**, Ileum **(H)**, Caecum **(I)** were purified and the presence of *S*. Enteritidis was identified, the number of copies for each sequence was normalized per μl of gDNA. The assay was performed in triplicate and included 4 to 6 chickens per group for each time point post-infection, except the bacterial load from liver that included 3 birds at 21 dpi. *p < 0.05, **p < 0.01, ***p < 0.001. Significance was determined using 2-way ANOVA with Sidak’s multiple comparisons, with 95% confidence interval.

To support the notion that the chickens with low H/L ratio are more resistant to *Salmonella* infection, 360 Jingxing Yellow chicks of one day old were dived in high and low H/L ratio groups based on their H/L ratio. The low H/L ratio chickens group was characterized by a significantly decreased H/L ratio (0.16 ± 0.03) as compared to the high H/L ratio chickens group (0.40 ± 0.09) (**Supplementary Table 2**). The survival rate and live weight post-infection of high and low H/L ratio SE-infected chickens were evaluated (**Figures 1A, B**). Remarkably, while only 83.96% of high H/L ratio chickens survived the oral SE infection, 97.50% of low H/L ratio individuals survived (**Figure 1A**, **Supplementary Table 3**). In addition, low H/L ratio chickens exhibited significantly reduced weight loss at 21 dpi than high H/L ratio chickens (**Figure 1B**). Further, we compared IL-1β, IL-8, and IFN-γ blood serum concentration between low and high H/L ratio chicken groups at 7 and 21 dpi (**Figure 1**). The concentration of IL-1β, IL-8, and IFN-γ was significantly increased in the serum of low H/L ratio chickens, compared to high H/L ratio chickens (**Figures 1C–E**). Moreover, we assessed blood, liver, ileum and caecum SE load between low and high H/L ratio chickens at 7 and 21 dpi. The bacterial load in the blood showed no significant difference between low and high H/L ratio chickens (**Figure 1F**). However, we found that compared to high H/L ratio chickens, low H/L ratio chickens exhibited a significantly lower liver (at 7 dpi, p < 0.05), ileum (at 21 dpi, p < 0.01), and caecum (at 7 dpi, p < 0.01) bacterial load during the infective cycle (**Figures 1G–I**).

**Figure 2:**
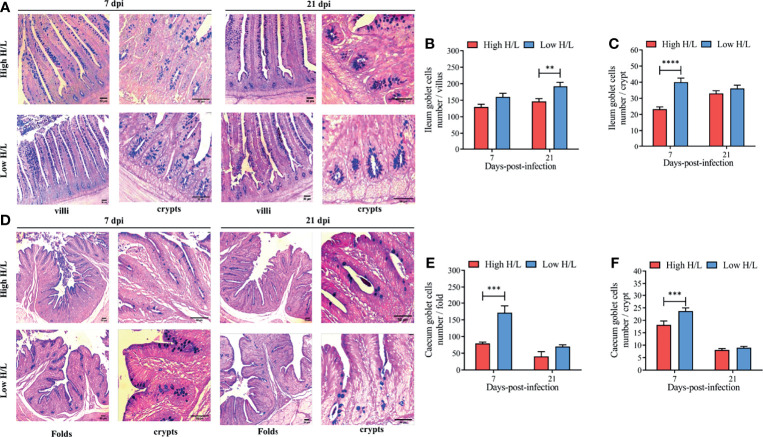
Low H/L ratio chickens exhibit higher ileal and cecal goblet cells number under S. Enteritidis infection at 7 and 21 dpi. **(A)** ileum villi and crypt goblet cells distribution at 7 and 21 dpi; **(B)** Ileum goblet cells number in the villi at 7 and 21 dpi; **(C)** Ileum goblet cells number in the crypts at 7 and 21 dpi; **(D)** Caecum folds and crypt goblet cells number distribution at 7 and 21 dpi; **(E)** Caecum goblet cells number in the folds at 7 and 21 dpi. **(F)** Caecum goblet cells number in the crypts at 7 and 21 dpi; **p < 0.01, ***p < 0.001, ****p < 0.0001. Data analysis was performed using 2-way ANOVA with Sidak’s multiple comparisons, with alpha = 0.05. Images were captured at 100X magnification (n = 2; 10 villi and 20 crypts each bird).

To evaluate the effects of SE infection on the goblet cell dynamics (number per villi, folds, and crypts) from the ileum and caecum, between low and high H/L ratio chickens at 7 and 21 dpi, we enumerated the number of goblet cells (**Figure 2**). **Figure 2A** presents the distribution of goblets cells along the ileal villi and crypts of low and high H/L ratio SE-infected chickens at 7 and 21 dpi. The results showed that under SE infection, the chickens with low H/L ratio exhibited significantly increased goblet cells number in the ileal villi (at 21 dpi, p < 0.01) and crypt (at 7 dpi, p < 0.0001), compared to the chickens with high H/L ratio (**Figures 2B, C**, respectively). The distribution of goblets cells along the folds and crypts in the caecum of low and high H/L ratio chickens at 7 and 21 dpi are presented in **Figure 2D**. The enumeration of goblet cells from the caecum also revealed that the chickens with low H/L ratio exhibited significantly increased (at 7 dpi, p < 0.001) goblet cells number in the cecal folds (**Figure 2E**) and crypt (**Figure 2F**), compared to the chickens with high H/L ratio.

**Figure 3:**
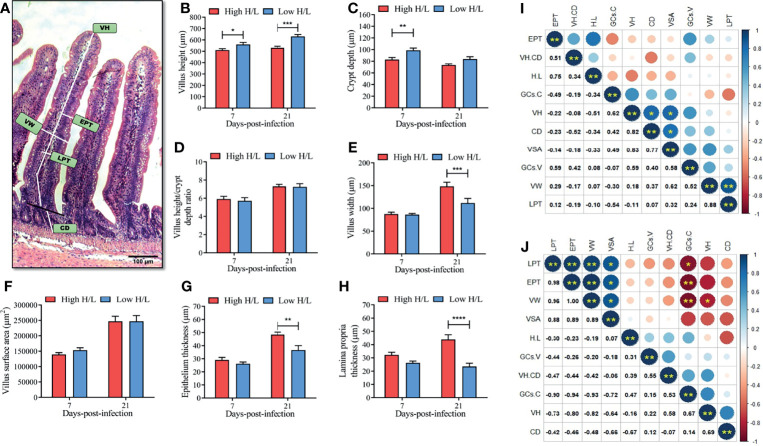
Ileal villi morphometry differences between high and low H/L ratio chickens at 7 and 21 days after S. Enteritidis infection. At 7 and 21 dpi, sections of 5µm of thickness from the ileum stained using hematoxylin and eosin (HE) were used for intestinal villi morphometry (n = 2; 10 villi and 20 crypts each bird); **(A)** ileal villi morphometry indexes; **(B)** villus height (VH) difference between low and high H/L ratio chickens SE-infected at 7 and 21 dpi; **(C)** Crypt depth (CD) difference between low and high H/L ratio chickens SE-infected at 7 and 21 dpi; **(D)** Villus height/crypt depth ratio (VH/CD) difference between low and high H/L ratio chickens SE-infected at 7 and 21 dpi; **(E)** villus width (VW) difference between low and high H/L ratio chickens SE-infected at 7 and 21 dpi; **(F)** villus surface era = π × villus height × villus width (VSA) difference between low and high H/L ratio chickens SE-infected at 7 and 21 dpi; **(G)** Epithelium thickness (EPT) difference between low and high H/L ratio chickens SE-infected at 7 and 21 dpi; **(H)** Lamina propria thickness (LPT) difference between low and high H/L ratio chickens SE-infected at 7 and 21 dpi; **(I, J)** spearman correlation between the different parameters related to the intestinal immune barrier function at 7 and 21 dpi, respectively; *p < 0.05, **p < 0.01, ***p < 0.001, ****p < 0.0001. Data analysis was performed using 2-way ANOVA with Sidak’s multiple comparisons, with alpha = 0.05.

To determine whether chickens with low or high H/L ratio possess enhanced intestinal barrier, we determined the ileal villi morphometry, and we assessed the correlation among the H/L and different histomorphometric indexes at 7 and 21 dpi (**Figure 3**). **Figure 3A** presents the different ileal villi morphological indexes measured using HE microscopic slides of the ileum section. The results showed no significant difference between low and high H/L ratio chickens in VH/CD (**Figure 3D**) and VSA (**Figure 3F**) at 7 and 21 dpi. However, we found that low H/L ratio chickens displayed greater ileal villi integrity, through higher villus height [VH, at 7 and 21 dpi, p < 0.05 and p < 0.001 respectively, (**Figure 3B**)], crypt depth (CD, at 7 dpi, p < 0.01, **Figure 3C**). Moreover, the results also showed that the chickens with low H/L ratio exhibited significantly reduced villus width (VW, p < 0.001), epithelium thickness (EPT, p < 0.01), and lamina propria thickness (LPT, p < 0.0001), compared to the chickens with high H/L ratio at 21 dpi (**Figures 3E, G, H** respectively). The correlation among H/L, the intestinal villi histomorphometric indexes, and goblet cells number in the villi and crypts (GCs.V and GCs.C respectively) from the ileum of low and high H/L ratio SE-infected chickens at 7 and 21 dpi are presented in the **Figures 3I, J** respectively. At 7 dpi, we observed that VH, was positively correlated with CD (r = 0.82, p < 0.05) and VSA (r = 0.83, p < 0.05, **Figure 3I**); that VW was positively correlated with LPT (r = 0.88, p < 0.01, **Figure 3I**). Remarkably, we found that H/L ratio was negatively correlated with VH (at 7 dpi) and CD (at 21 dpi) (**Figure 3I**). At 21 dpi, we found that EPT, LPT, VSA and VW were positively correlated (**Figure 3J**). While, GCs.C, VH and CD were negatively correlated with LPT, EPT, VW and VSA (**Figure 3I**).

### The H/L Ratio Affects the Cecal SCFAs Content During S. Enteritidis Infection

To assess the differences in SCFAs cecal contents between low and high H/L ratio SE-infected chickens, cecal contents were sampled and submitted to Gas Chromatography-Mass Spectrometry (GC-MS) analysis. The SCFAs contents differences between low and high H/L chickens under *Salmonella* at 7 and 21 dpi are shown in **Table 1**. It was noteworthy that cecal butyrate, hexanoate, isovalerate, and iso-hexanoate did not present a significant difference between the two groups over the two-time points post-infection. However, low H/L ratio chickens exhibited significantly increased cecal acetate levels at 7 and 21 dpi, compared to high H/L ratio chickens (p < 0.05). At 21 dpi, propionate cecal content was significantly higher in low H/L ratio chickens than high H/L ratio chickens (p < 0.05). Furthermore, at 7 dpi, the low H/L ratio chickens showed significantly increased valerate and iso-butyrate, compared to high H/L ratio chickens (p < 0.05 and p < 0.01, respectively).

**Table 1:** Effects of Heterophil/Lymphocyte ratio level on cecal SCFA contents of SE-infected chickens at 7 and 21 days post-infection (mmol/kg).

Items	Groups					
	SE-infected 7 dpi			SE-infected 21 dpi		
	High H/L	Low H/L	*p*-value	High H/L	Low H/L	*p*-value
Acetate	7.29 ± 3.31	13.65 ± 1.89	**0.0001**	11.36 ± 1.50	15.93 ± 4.57	**0.033**
Propionate	0.88 ± 0.14	1.18 ± 0.54	0.1134	6.26 ± 1.43	9.03 ± 2.55	**0.0241**
Butyrate	4.99 ± 1.72	5.06 ± 2.36	0.4735	6.00 ± 1.31	6.73 ± 2.22	0.2719
Valerate	0.04 ± 0.03	0.09 ± 0.04	**0.0191**	1.48 ± 0.32	1.53 ± 0.33	0.3897
Hexanoate	0.035 ± 0.030	0.038 ± 0.028	0.4382	0.044 ± 0.023	0.037 ± 0.024	0.2942
Iso-butyrate	0.14 ± 0.12	0.44 ± 0.26	**0.0082**	0.47 ± 0.18	0.60 ± 0.36	0.2176
Isovalerate	0.16 ± 0.16	0.26 ± 0.15	0.1583	0.28 ± 0.10	0.36 ± 0.29	0.3431
Iso-hexanoate	0.008 ± 0.008	0.015 ± 0.022	0.2804	0.006 ± 0.00	0.007 ± 0.001	0.0767
*Total SCFAs	13.54 ± 2.83	20.73 ± 4.78	0.327	25.90 ± 4.19	34.22 ± 5.82	0.3438

five to six birds were used for SCFAs analysis, except for the isovalerate and isohexanoate, which 3 biological replicates were used for statistical analysis.

Bold means significantly different (p-values < 0.05). *Sum of of the SCFAs.

### 16S rRNA Gene Sequencing Confirmed Differential Gut Microbiota Composition, and Metagenomic Sequencing Revealed the Potential Bacterial Taxa Involved in the Resistance Against S. Enteritidis Infection in Chicken

**Figure 4:**
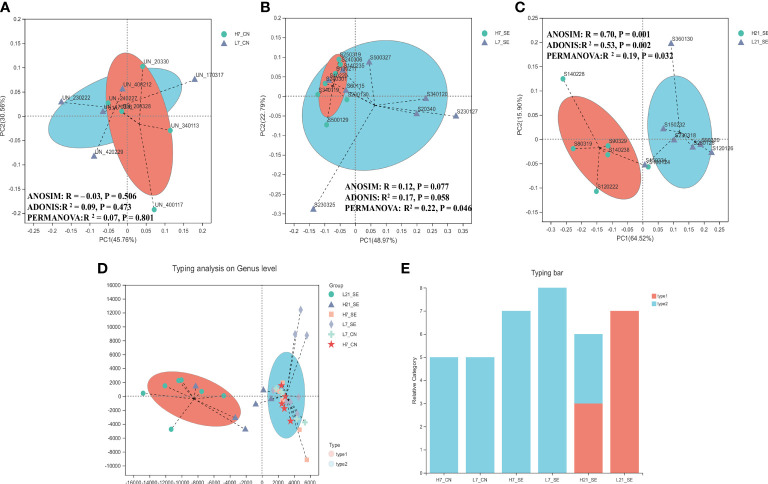
Comparison analysis of cecum microbiota profile of non-infected SE-infected high and low H/L chickens. **(A)** Beta diversity, Principal coordinates analysis (PCoA) performed with weighted UniFrac distances showed no clear separation pattern between low and high H/L ratio non-infected chickens at 7 dpi. **(B)** Beta diversity, Principal coordinates analysis (PCoA) performed with weighted UniFrac distances showed no clear separation pattern between low and high H/L ratio SE-infected chickens at 7 dpi. **(C)** beta diversity, Principal coordinates analysis (PCoA) performed with weighted UniFrac distances showed more visible separation of clustering pattern between low and high H/L ratio SE-infected chickens at 21 dpi. **(D)** Typing analysis on genus level showing samples clustering according dominant species. **(E)** Bar typing analysis showing distribution of dominant species among groups. H7_CN: high H/L non-infected 7 dpi (n = 5); L7_CN: low H/L non-infected 7 dpi (n = 5); H7_SE: High H/L SE-infected 7 dpi (n = 8); L7_SE: Low H/L SE-infected 7 dpi (n = 7); H21_SE: high H/L SE-infected 21 dpi (n = 6); L21_SE: Low H/L SE-infected 21 dpi (n = 7).

The 16S rRNA gene sequencing of cecal contents of high and low H/L ratio chickens were analyzed at 7 and 21 days after SE infection to assess the cecal microbiome composition. The diversity data analysis of all the samples generated a total of optimized sequence ranging between 2895720 and 1189879657 bases, with an average sequence length of 411bp. The species annotation showed a total of 740 ASVs including 17 phyla, 23 classes, 49 order, 83 families, 165 genera, and 225 species, with 113 ASVs and 47 genera shared among all groups (**Supplementary Figures 2A, B**, respectively). The diversity indexes (Shannon and Simpson) showed significant (p = 0.0034 and p = 0.0053, respectively) differences between high and low H/L ratio chickens at 21 dpi (**Table 2**). However, the alpha diversity richness index Chao showed no significant difference between the two groups at 7 and 21 dpi (**Table 2**). No significant differences were observed between high and low H/L ratio non-infected chickens at 7 dpi in terms of Shannon, Simpson and Chao indexes (**Table 2**). Principal Coordinates Analysis (PCoA) with weighted UniFrac distances measured the phylogenetic similarities and relative abundance of OTUs shared between high and low H/L ratio chickens (with corresponding R and p-value reported). The PCoA showed that high and low H/L ratio chickens display less visible clustering patterns separation at 7 dpi under normal and SE infection conditions (**Figures 4A, B**, **Supplementary Table 5** for beta diversity statistics). However, as the infection time progressed, a separation between two groups became more apparent with a significant increase in R-value from 7 dpi (R^2^ = 0.17, p = 0.058) to 21 dpi (R^2^ = 0.53, p = 0.002) based on Weighted UniFrac analysis (**Supplementary Table 5**). PERMANOVA analysis for group as factor revealed also a significant increase in R-value from 7 dpi (R^2^ = 0.056, p = 0.587) to 21 dpi (R^2^ = 0.27, p = 0.008) (**Supplementary Table 6**), while for the H/L ratio as grouping factor the results revealed a decrease in R-value from 7 dpi (R^2^ = 0.22, p = 0.046) to 21 dpi (R^2^ = 0.19, p = 0.032) (**Figures 4B, C**). To classify the cecal microbiota of high and low H/L ratio chickens at 7 and 21 dpi, a microbiota typing analysis was performed among the six groups. Through this analysis the samples and groups with similar dominant cecal flora were clustered into categories (type 1 and type 2) in order to identify the major bacterial taxa dominant in high and low H/L ratio chickens under normal and infected conditions (**Figures 4D, E**). The typing analysis on genus level showed 2 major cluster, the type 1 and type 2 categories. The top 3 bacterial species of type 1 category were *Bacteroides* (36.18%), unclassified *Lachnospiraceae* (15.61%) and *Lactobacillus* (5.42%), while the type 2 category included unclassified *Lachnospiraceae* (26.54%)*, Escherichia-Shigella* (9.43%) and *Ruminococcus torques* group (7.28%).

**Table 2:** Alpha diversity (genus level) of cecal microbiota of Control (7dpi) and SE-infected (7 and 21 dpi) High and Low H/L ratio chicken groups.

Items	Groups								
	Control (7 dpi)			SE-infected 7 dpi			SE-infected 21 dpi		
	H7_CN	L7_CN	*p*-value	H7_SE	L7_SE	*p*-value	H21_SE	L21_SE	*p*-value
**Chao**	47.4 ± 5.81	43.4 ± 14.77	0.5309	44.14 ± 9.21	47 ± 7.90	0.4511	63.5 ± 10.17	59.86 ± 3.40	0.5174
**Shannon**	2.52 ± 0.27	2.19 ± 0.78	0.6761	2.51 ± 0.39	2.34 ± 0.53	0.6025	2.78 ± 0.08	2.30 ± 0.20	**0.0034**
**Simpson**	0.16 ± 0.05	0.22 ± 0.15	0.6761	0.15 ± 0.10	0.20 ± 0.11	0.3854	0.12 ± 0.02	0.23 ± 0.06	**0.0053**

H7_CN: high H/L non-infected 7 dpi (n = 5); L7_CN: low H/L non-infected 7 dpi (n = 5); H7_SE: High H/L SE-infected 7 dpi (n = 8); L7_SE: Low H/L SE-infected 7 dpi (n = 7); H21_SE: high H/L SE-infected 21 dpi (n = 6); L21_SE: Low H/L SE-infected 21 dpi (n = 7).

Bold means significantly different (p-values < 0.05).

**Figure 5:**
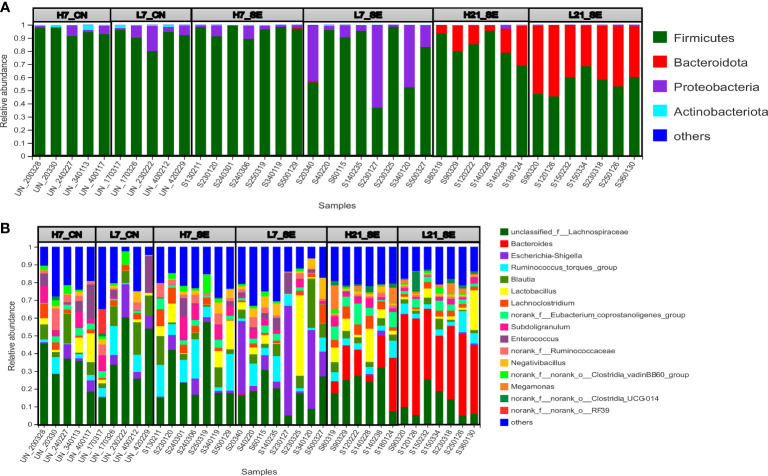
Relative abundance of cecal microbiota phyla and genera of high and low H/L ratio SE-infected chickens at 7 and 21 dpi, by 16s profiling. **(A)** Relative abundance at phylum level. **(B)** Relative abundance at genus levels. H7_CN: high H/L non-infected 7 dpi (n = 5); L7_CN: low H/L non-infected 7 dpi (n = 5); H7_SE: High H/L SE-infected 7 dpi (n = 8); L7_SE: Low H/L SE-infected 7 dpi (n = 7); H21_SE: high H/L SE-infected 21 dpi (n = 6); L21_SE: Low H/L SE-infected 21 dpi (n = 7).

A community bar plot was used to illustrate the relative abundance at each level, and identify differentially abundant taxonomic features at the phylum and genus levels. In the present study, the more abundant phyla were *Firmicutes*, *Bacteroidetes*, *Proteobacteria* and *Actinobacteria*, with a differential abundance among groups, treatment and over time points post-infection (**Figure 5A**). Under SE infection at 7 dpi, we observed that low H/L ratio chickens displayed an abundance in *Firmicutes* (78.08%), *Bacteroidetes* (0.14%), *Proteobacteria* (21.37%) and *Actinobacteria* (0.32%) different from high H/L ratio chickens (95.72, 0.10, 3.67 and 0.50%, respectively) (**Figure 5A**). At 21 dpi, low H/L ratio chickens exhibited an abundance in *Firmicutes* (56.31%), *Bacteroidetes* (43.12%), *Proteobacteria* (0.40%) and *Actinobacteria* (0.13%) different from high H/L ratio chickens (81.84, 17.05, 0.84% and 0.21, respectively) (**Figure 5A**). Based on Kruskal-Wallis H test, the six groups displayed significantly different *Firmicutes* (p = 0.0018), *Bacteroidetes* (p = 0.000024), *Proteobacteria* (p = 0.00051) and *Actinobacteria* (p = 0.000248) relative abundance (**Figure 5A**).

**Figure 6:**
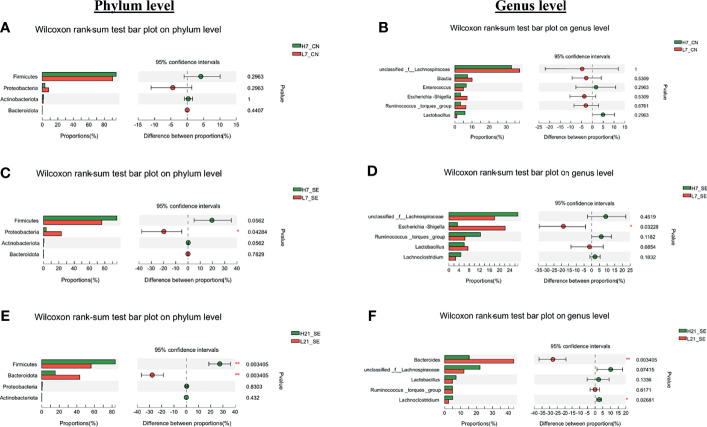
Comparative analysis of relative abundance of cecal microbiota phyla and genera. **(A)** Cecal microbiota phyla differential abundance between high and low H/L ratio non-infected chickens at 7 dpi. **(B)** Cecal microbiota genera differential abundance between high and low H/L ratio non-infected chickens at 7 dpi. **(C)** Cecal microbiota phyla differential abundance between high and low H/L ratio SE-infected chickens at 7 dpi. **(D)** Cecal microbiota genera differential abundance between high and low H/L ratio SE-infected chickens at 7 dpi. **(E)** Cecal microbiota phyla differential abundance between high and low H/L ratio SE-infected chickens at 21 dpi. **(F)** Cecal microbiota genera differential abundance between high and low H/L ratio SE-infected chickens at 21 dpi. Data analyzed by Wilcoxon rank-sum test, with reported p-value and significances. H7_CN: high H/L non-infected 7 dpi (n = 5); L7_CN: low H/L non-infected 7 dpi (n = 5); H7_SE: High H/L SE-infected 7 dpi (n = 8); L7_SE: Low H/L SE-infected 7 dpi (n = 7); H21_SE: high H/L SE-infected 21 dpi (n = 6); L21_SE: Low H/L SE-infected 21 dpi (n = 7).

From the 165 bacterial genera identified in this work, an unclassified bacterium from the family of *Lachnospiraceae*, *Bacteroides*, *Escherichia-shigella*, *Ruminococcus torques*, *Blautia*, and *Lactobacillus* were the more abundant (**Figure 5B**). The comparative analysis of the relative abundance on the genus level showed that only the unclassified bacterium from the family of *Lachnospiraceae* (p = 0.02098), *Bacteroides* (p = 0.0000058) and *Escherichia-shigella* (p = 0.00104) were significantly different among the six groups (**Figure 5B**). Next, we performed a comparative analysis between low and high H/L ratio chickens cecal microbiota relative abundance at the phylum and genus level (**Figure 6**). It was noteworthy that despite a differential cecal flora structure, no significant difference on phylum and genus level were observed between high and low H/L ratio chickens under normal condition at 7 dpi (**Figures 6A, B**). However, at 7 dpi, the two groups’ relative abundance comparison at the phylum and genus level showed a significant difference in *Proteobacteria* and *Escherichia-shigella* (p = 0.04284 and p = 0.03228, respectively) between low and high H/L ratio SE-infected chickens (**Figures 6C, D**). While, at 21 dpi, low H/L ratio chickens exhibited an abundance in *Firmicutes* (56.31%), *Bacteroidetes* (43.12%), *Proteobacteria* (0.40%) and *Actinobacteria* (0.13%) different from high H/L ratio chickens (81.84, 17.05, 0.84% and 0.21, respectively) (**Figure 5A**). Moreover, low H/L ratio chickens exhibited significantly increased *Bacteroidetes* and significantly decreased *Firmicutes* abundance at the phylum level (p = 0.003405), while on the genus level we found significantly increased *Bacteroides* and significantly decreased *Lachnoclostridium* (p = 0.003405 and p = 0.02681, respectively; **Figures 6E, F**).

A comparative analysis of the relative abundance at the phylum and genus level between non-infected (CN) and SE-infected (SE) for a given H/L ratio at 7 dpi was performed (**Supplementary Figure 3**). The results showed no significant differences between the CN and SE groups at 7 dpi for a given H/L group, except for low H/L ratio at the phylum level. We found that low H/L ratio chickens SE-infected displayed significantly (p = 0.015) reduced *Actinobacteria*, compared to low H/L ratio chickens non-infected chickens (**Supplementary Figure 3C**).

Based on the metagenomic sequencing, the bacterial taxa identified by 16S rRNA were confirmed, and new bacterial taxa were identified as significantly different between low and high H/L chickens infected by *Salmonella* Enteritidis. Moreover, the species *Bacteroides plebeius* was identified as a potential host immunomodulator during *Salmonella* infection in chicken, which can play a protective role (**Supplementary Figure 4**).

### Correlations Among Cecal Microbiota Genus, H/L, and SCFAs

**Figure 7:**
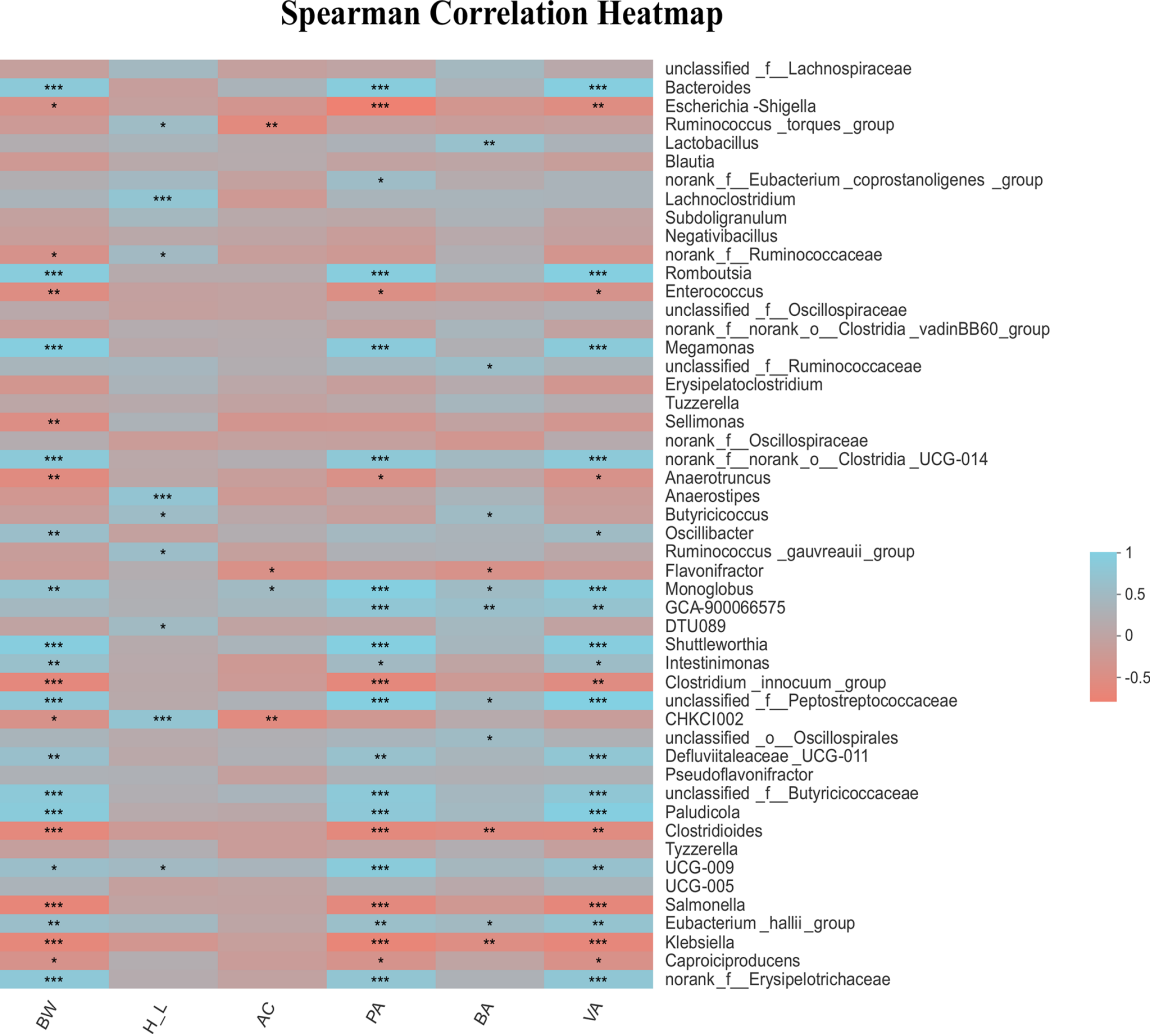
Correlation of cecal microbiota genus with body weight post-infection, H/L ratio and SCFAs concentration in the caecum of SE-infected chickens at 7 and 21 dpi. Turquoise positive correlation; Salmon, negative correlation; *strong correlation (|R| > 0.55, p < 0.05). BW: body weight post-infection; H_L: heterophil/lymphocyte ratio; AC: acetate; PA: propionate; BA: butyrate; VA: valerate. In total 28 biological replicates were used to perform this analysis, including H7_SE: High H/L SE-infected 7 dpi (n = 8), L7_SE: Low H/L SE-infected 7 dpi (n = 7), H21_SE: high H/L SE-infected 21 dpi (n = 6) and L21_SE: Low H/L SE-infected 21 dpi (n = 7). *0.01 < P ≤ 0.05, **0.001 < P ≤ 0.01, ***P ≤ 0.001.

In the current work the difference in microbiota profile between low and high H/L ratio chickens was mainly found at 21 dpi. The major bacteria phyla were *Firmicutes*, *Bacteroidetes* and *Proteobacteria* at 7 and 21 dpi. The association among cecal microbiota genera, body weight post-infection, H/L and SCFAs was further analyzed by spearman correlation analysis and illustrated by heatmap (**Figure 7**). The results showed that a bacterial genus such as *Bacteroides* was significantly and positively correlated with the body weight post-infection (r = 0.72, p = 3.09E-05; **Figure 7**). Moreover, we found that the H/L ratio was significantly and positively correlated with bacterial genus such as *Anaerostipes* (r = 0.63, p = 0.00062), unclassified CHKCI002 (r = 0.61, p = 0.00081) and *Lachnoclostridium* (r = 0.63, p = 0.00056). It was noteworthy that we recorded several significant positive and negative correlations between the cecal microbiota genera and propionate and valerate cecal contents. Interestingly, *Bacteroides* was significantly and positively correlated with propionate (r = 0.78, p = 2.32E-06) and valerate (r = 0.82, p = 3.68E-07) contents, while *Salmonella* was significantly and negatively correlated with body weight post-infection (r = − 0.67, p = 0.00016), propionate (r = − 0.61, p = 0.00098) and valerate (r = − 0.65, p = 0.00036) contents (**Figure 7**). Furthermore, we observed that the genus *Escherichia shigella* was significantly and negatively correlated with propionate (r = − 0.78, p = 2.65E-06) and valerate (r = − 0.54, p = 0.0045) contents (**Figure 7**).

### Functional Capacity of Cecal Microbiota Based on Metagenomic Sequencing

Using metagenomic data, we investigated the functional capacity of the cecal microbiome of high and low H/L ratio chickens SE-infected. Annotation of ORFs predicted from assembled contigs revealed a total of 10,984,439 ORFs with an average length of 587.98 bp consistent with functional capacity. The predicted genes were classified by aligning them to the KEGG, CARD, and VFDB databases. **Supplementary Figures 5** and **6** present the shared and unique genes, the relative abundance in KEGG pathways, the virulence factors, and antibiotic resistance genes based on CARD between low and high H/L ratio SE-infected chickens at 7 and 21 dpi. In addition, we investigated the difference in gene number among H7, L7, H21, and L21 and found that the number of non-redundant genes is higher in L7 and L21 (chickens with low H/L ratio), compared to H7 and H21 (chickens with high H/L ratio). The numbers of shared and unique genes among the four groups were determined, we found that 1743 genes were common (**Supplementary Figure 5A**). The relative abundance in KEGG pathways level 1 was evaluated and represented by bar plot graphics (**Supplementary Figure 5B**). The results showed that at 7 dpi, chickens with low H/L ratio displayed more enriched pathways related to organismal systems and human disease, while high H/L ratio showed more enriched pathways related to the metabolism (**Supplementary Figure 5B**). The antibiotic resistance genes name and the virulence factors were also assessed (**Supplementary Figures 6A, B**).

**Figure 8:**
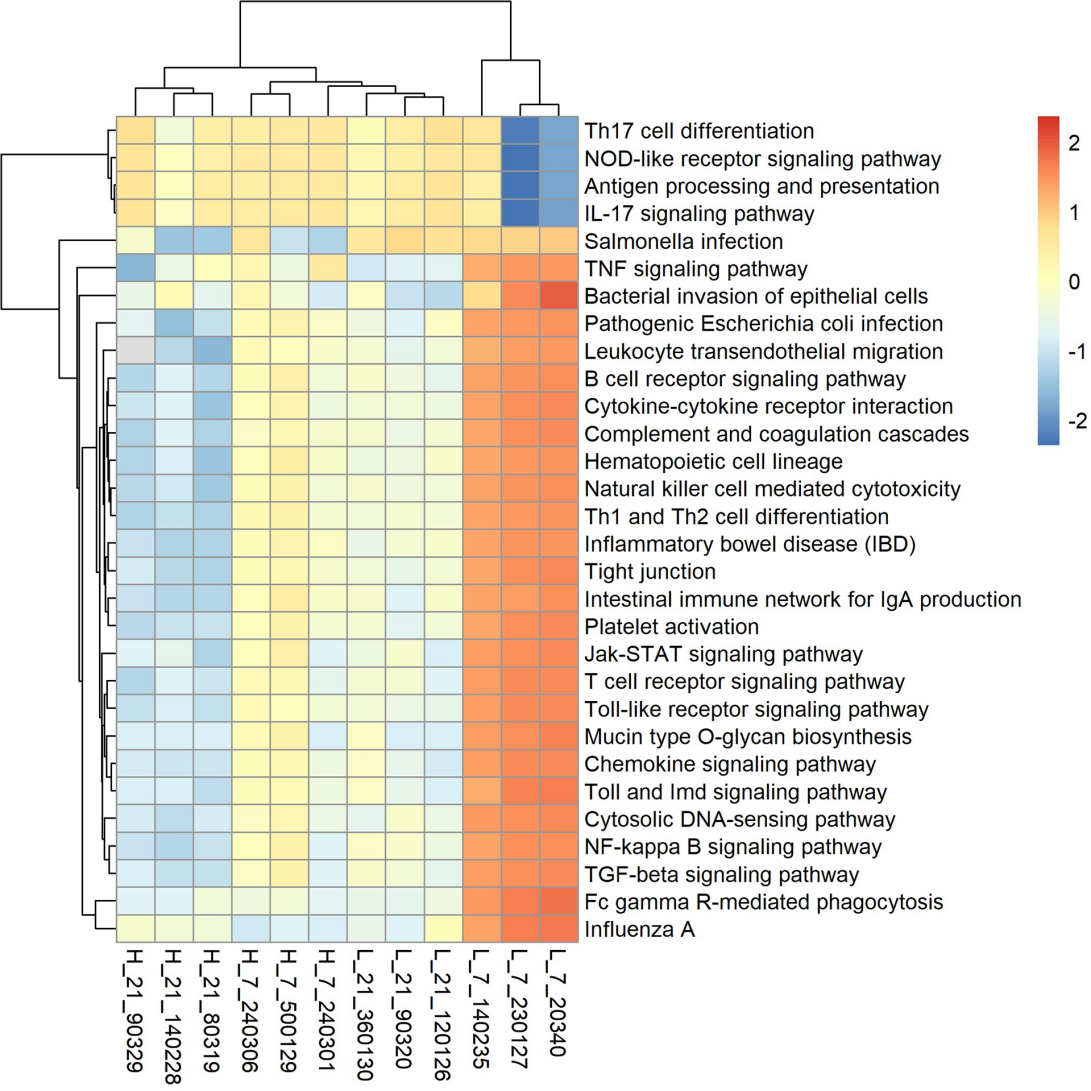
Heatmap analysis of KEGG pathways (level 3) of SE-infected high and low H/L chickens at 7 and 21 dpi (n = 3). Red, high KO abundance; blue, low KO abundance.

Based on the metagenomic sequencing, we used Lefse (linear discriminant analysis (LDA) effect size) analysis to discover differentially abundant pathways between low and high H/L ratio SE-infected chickens at 7 and 21 dpi (**Supplementary Figure 7**). This analysis showed that low H/L chickens possess more enriched pathways related to transport, immune disease and resistance, while high H/L chickens exhibit more enriched metabolic pathways (**Supplementary Figure 7**). We further identified 30 KOs (**Figure 8**) related to immune diseases, immune system, infectious diseases (bacterial and viral), glycan biosynthesis and metabolism, signal transduction, signaling molecules and interaction, and cell community-eukaryotes. Seven days after infection, the abundance of 25 KEGG pathways out of the 30, including *Salmonella* infection, TNF signaling pathway, bacterial invasion of epithelial cells, pathogenic *Escherichia coli* infection, Leukocyte transendoepithelial migration, cytokine-cytokine receptor interaction, complement and coagulation cascades, hematopoietic cell lineage, natural killer cell-mediated cytotoxicity, Th1 and Th2 cell differentiation, inflammatory bowel disease (IBD), tight junction, intestinal immune network for IgA production, platelet activation, jak-STAT signaling pathway, cytosolic DNA-sensing pathway, NF-κB signaling pathway, TGF-beta signaling pathway, toll-like receptor signaling pathway, T cell receptor signaling, B cell receptor signaling pathway, mucin type O-glycan biosynthesis, chemokine signaling pathway and Influenza A were significantly enriched in low H/L ratio chickens, compared to high H/L ratio chickens. However, 4 KEGG pathways, including Th17 cell differentiation, NOD-like receptor signaling pathway, antigen processing and presentation, and IL-17 signaling pathway, were more enriched in high H/L ratio chickens than low H/L ratio chickens at 7 dpi (**Figure 8**). Remarkably, we did not observe a significant difference in the abundance of enrichment of the 30 KOs at 21 dpi.

### Antibiotic Resistance Profiles of Resistant (Low H/L) and Susceptible (High H/L) Chickens Cecal Microbiome During S. Enteritidis Infection

To explore the resistome of high and low H/L ratio SE-infected chickens, we identified the antibiotic resistance genes (ARGs) present in their cecal microbiota. The metagenome data were examined for identification of antibiotic resistance factors genes (**Supplementary Figure 8**) using the Comprehensive Antibiotic Resistance Database (CARD) (**Table 3**). At the class level, the annotation was performed by choosing a relative abundance of antibiotic resistance genes > 1%. As result, 426 AROs were identified between high and low H/L ratio chickens 7 and 21 days after SE infection following the CARD annotation. At 7 dpi, the more abundant AROs identified were related to efflux pump, glycopeptide resistance gene, included ARO:3000535, ARO:3002987, ARO:3003948, ARO:3000816, and ARO:3003728 (**Table 3**). While at 21 dpi, they were related to efflux pump and antibiotic target protection protein, included: ARO:3000838, ARO:3000816, ARO:3003950, ARO:3000024, ARO:3002881, ARO:3003844 (**Table 3**).

**Table 3:** Top differentially abundant antibiotic resistance genes between high and low H/L ratio SE-infected chickens based on CARD database annotation.

Dpi	ARO_accession	ARO_name	ARO_description	High H/L	Low H/L	Total
7	ARO:3000535	macB	efflux pump conferring antibiotic resistance	186614.57	119323.33	152969
	ARO:3002987	bcrA	efflux pump conferring antibiotic resistance	129499.43	77335	103417
	ARO:3003948	efrA	efflux pump conferring antibiotic resistance	55466.28	37465	46465.6
	ARO:3000816	mtrA	efflux pump conferring antibiotic resistance; gene modulating antibiotic efflux	35186.57	21259.33	28223
	ARO:3003728	vanRI	glycopeptide resistance gene; gene conferring antibiotic resistance *via* molecular bypass; antibiotic resistance gene cluster, cassette, or operon	31387.14	19896.66	25641.9
21	ARO:3000838	arlR	efflux pump conferring antibiotic resistance; gene modulating antibiotic efflux	44618.66	33624.28	39121.5
	ARO:3000816	mtrA	efflux pump conferring antibiotic resistance; gene modulating antibiotic efflux	33085.66	22460.86	27773.3
	ARO:3003950	msbA	efflux pump conferring antibiotic resistance	66771.33	44274.57	55523
	ARO:3000024	patA	efflux pump conferring antibiotic resistance	27094.33	32503.43	29798.9
	ARO:3002881	lmrC	efflux pump conferring antibiotic resistance	51802	34890.28	43346.1
	ARO:3003844	mfd	antibiotic target protection protein; fluoroquinolone resistance gene	60226.33	58644	59435.2

Twenty-six birds were used, including H7: High H/L SE-infected 7 dpi (n = 7), L7: Low H/L SE-infected 7 dpi (n = 6), H21: high H/L SE-infected 21 dpi (n = 6) and L21: Low H/L SE-infected 21 dpi (n = 7).

The contribution of cecal microbiota genera to the AGRs and virulence factors were also assessed (**Supplementary Figures 8** and **9**, respectively). The species and functional annotation analysis revealed that *Clostridium*, *Bacteroides*, *Blautia*, and *Escherichia coli* in descending order of contribution were the bacteria for which the functional contribution was the most enriched.

### Virulence Factors Profiles of Resistant (Low H/L) and Susceptible (High H/L) Chickens Cecal Microbiome During S. Enteritidis Infection

Metagenomic data were screened against the Virulence Factors Database (VFDB) to investigate the abundance of virulence factors. The annotation of virulence factors between low and high H/L ratio chickens SE-infected was performed 7 and 21 days after infection. The results showed that low H/L ratio chickens displayed lower virulence factors abundance, compared to high H/L ratio chickens. The functional classification at level 1 revealed that the predominated virulence factors identified in this study were related to defensive virulence factors (antiphagocytosis), regulation of virulence-associated genes (regulation), non-specific virulence factors (iron uptake system), and offensive virulence factors (secretion system and adherence). Moreover, they varied among the chicken groups and overtime points post-infection, which could explain the differential *Salmonella* resistance and fecal microbiome composition. The main significantly differentially abundant virulence factors, their function, and the involved species are presented in **Table 4**.

**Table 4:** Top differentially abundant virulence factors between high and low H/L ratio SE-infected chickens based on VFDB database annotation.

Dpi	Level 1	Level 2	VFs	Function	Species
7	Defensive virulence factors	Antiphagocytosis	Capsule	glycosyl transferase, group 2 family protein	*E.* Faecalis
	Regulation of virulence-associated genes	Regulation	PhoP	Possible two component system response transcriptional positive regulator PhoP	*M. Tuberculosis*
	Nonspecific virulence factor	Iron uptake system	FbpABC	Iron (III) ABC transporter, ATP-binding protein	*N*. *meningitidis*
	Offensive virulence factors	Secretion system	HSI-I	serine/threonine protein kinase PpkA	*P. aeruginosa*
21	Defensive virulence factors	Antiphagocytosis	Alignate	alginate biosynthesis regulatory protein AlgR	*P*. *aeruginosa*
	Nonspecific virulence factor	Iron uptake system	FbpABC	Iron (III) ABC transporter, ATP-binding protein	*N. meningitidis*
	Regulation of virulence-associated genes	Regulation	PhoP/R	Possible two component system response transcriptional positive regulator PhoP	*M. tuberculosis*
	Offensive virulence factors	Adherence	Type IV pili	two-component response regulator PilR	*P. aeruginosa*
	ND	ND	Cytolysin	ND	ND

Twenty-six birds were used, including H7: High H/L SE-infected 7 dpi (n = 7), L7: Low H/L SE-infected 7 dpi (n = 6), H21: high H/L SE-infected 21 dpi (n = 6) and L21: Low H/L SE-infected 21 dpi (n = 7). ND, Not determined.

## Discussion

It is unknown how the host immune cells interact with the intestinal microbiome. Furthermore, how commensal bacteria interact with host immune cells to shape and control the immune system remains unclear. The gastrointestinal tract represents an important location for the development of immune cells, which regulate microbial diversity and maintain extra-intestinal immunity (45). This study compared the disease resistance, intestinal immune barrier function, SCFAs contents, cecal microbiome composition, resistome, and virulome of high and low H/L ratio SE-infected chickens. Our results demonstrated that the chickens with low H/L ratio are more resistant to SE and possess an enhanced immune response than the high H/L ratio chickens. Moreover, we found that low H/L ratio chickens exhibited significantly higher intestinal immune barrier function through increased abundance in goblet cells and enhanced intestinal villi morphology. Furthermore, we observed that the composition of the cecal microbiota of low H/L ratio chickens differed significantly from that of the high H/L ratio chickens at 7 and 21 days after SE infection. However, the findings of this study have to be seen in light of some limitations.

Recent researches have established the critical roles of gut microbiota and SCFAs in developing immunity against harmful bacterial pathogens in poultry. Commensal bacteria protect against pathogenic invasion by directly competing with them, producing antibodies, and stimulating the production of various cytokines that modulate innate and adaptive immunity. Heterophil and lymphocyte cells are the two more abundant white blood cells playing a significant role in innate and adaptative immunity, respectively (46). Heterophils form the prime line of immune protection against bacterial pathogens in inflammatory lesions. However, the lymphocytes cells play a central role in humoral adaptative immunity (B cells) and cell-mediated adaptative immunity (T cells) (21). The Heterophil/Lymphocyte (H/L) ratio is an essential immune and stress indicator in chicken breeding. A low H/L ratio is an ancestral state in birds, which may provide a long life span and survival (21).

Moreover, low H/L chickens exhibited resistance advantages such as survival, reduced body weight loss and SE load in different organs and tissues, enhanced cytokines and chemokines blood serum concentrations. These observations suggest that low H/L ratio chickens are more resistant to *Salmonella* infection than high H/L ratio chickens. These results are in accordance with the previous studies (21, 26, 47, 48). The H/L ratio is an immune feature that can reflect the resistance and immune response capacity. Birds with a low H/L ratio have enhanced resistance to heat stress (49, 50). Low H/L ratio is used as selection criteria for *Salmonella* Typhimurium resistance (24). Furthermore, the same authors have proved that the H/L ratios are heritable in chickens.

Mucosal immunity constitutes the first component in host defense against pathogens (51). The intestinal mucosa forms a barrier that encompasses mechanical, immune, and biological barriers. In addition, they maintain intestinal homeostasis, prevent the displacement of endotoxins, limit the invasion of pathogens and regulate the microbial-host immune response. Goblet cells can deal rapidly with the pathogens suspected to cause intestinal infection by infiltrating the epithelium layer through non-specific endocytosis and secreting mucus (52). In this process, goblet cells die in playing a crucial role in protecting intestinal mucosa and epithelium (53). Our study observed that the low H/L ratio chickens displayed significantly higher abundance in goblet cells than high H/L ratio chickens. This result suggests that the chickens with low H/L ratio display an enhanced intestinal barrier. A high number of lymphocyte cells, characteristic of a low H/L, could be the reason for this enhanced intestinal immune barrier. T lymphocytes can defend the host by removing infected cells or recruiting other immuno-protective and immune regulatory cells (54).

After *Salmonella* infection, several histopathologic alterations occur when a pathogen breaks the mucus barrier, colonizes, and invades the intestinal epithelium (55). The main symptoms of Salmonellosis occur during the first 7 days post-infection. *Salmonella* destroys and invades the intestinal epithelial tissue during this period, and the host immune system fights to protect and clear this pathogen (56). The present work showed that chickens with low H/L ratio displayed increased VH, CD and VSA, while chickens with high H/L ratio exhibited increased EPT and LPT. These results indicate that chickens with low H/L ratio were less susceptible to SE infection. In accordance with our results, Fasina and collaborators (55) reported a substantial increase of the LPT in two studies evaluating the influence of S. Typhimurium infection on intestinal goblet cell dynamics (density and size) and villus morphology in broiler chicks. The mucus produced by the intestinal goblet cells acts as an important barrier against ST invasion (57, 58). IFN-γ, produced by T lymphocytes and natural killer cells, is a pleiotropic chemical that has a distinct effect on each step of the immune response. (59–62). Several previous studies have demonstrated that the rate of *Salmonella* infection clearance corresponds with an increase in IFN-γ mRNA expression and a robust T cell response (63, 64). These results taken together suggest that it is possible that the chickens with low H/L ratio display enhanced immune cells function, compared to the chickens with high H/L ratio.

In this study, *Salmonella* infection induced significant changes of the microbiome composition, and phenotypic differences between individuals with high and low H/L ratio were observed. In contrast, Mon and colleagues (29) observed no significant difference in the bacterial load from the caecum using the plate method and no bacteria were detected in the immune organs (liver and spleen). Moreover, according to their results and the age of the chickens at the moment of the infection (14 days old), they obtained no significant difference 3 days after infection in terms of microbiota composition (29). The results obtained in this study may be attributed to the dosage of *Salmonella* and the age of infection (7 days old). It is possible to suggest that such inoculum dosage potentially can show earlier changes in microbiome composition and may induce a strong immune response that will not only detect *Salmonella* in the immune organs but also display phenotypic differences among individuals.

The microbiota and its metabolites can influence host physiology and pathophysiology by regulating various metabolic, inflammatory, and even behavioral processes (13, 18, 19). This work observed that low H/L ratio chickens displayed enhanced acetate, propionate, valerate, and iso-butyrate levels under *Salmonella* infection compared to high H/L ratio chickens. Our results also revealed that acetate and propionate were the main cecal SCFAs. These SCFAs can reduce intestinal inflammation when the butyrate is scarce due to the decrease in butyrate producer bacteria caused by *Salmonella* infection. They can also create an acidic environment in the gastrointestinal tract to restrain pathogens’ growth and invasion (65). Intestinal epithelial cells can consume acetate and propionate and have diverse anti-inflammatory effects (66, 67). They can inhibit the production of proinflammatory cytokines induced by TLR4 stimulation. Similar to butyrate, propionate can induce T cells to differentiate into T regulatory cells (67, 68). Acetate can regulate intestinal inflammation by the stimulation of G protein-coupled receptors 43 (GPR43) (69), maintaining the epithelial barrier function (70). SCFAs-mediated GPR43 is essential for reducing some inflammatory responses (70). Maslowski and colleagues (70) suggested that the SCFAs and GPR43 interactions are molecular links connecting microbial metabolites, host immune systems, and inflammatory responses. Acetate possesses anti-inflammatory effects in neutrophils, which is the equivalent of heterophil cells in avian, by inhibiting NF-κB activation by decreasing the level of proinflammatory mediators such as lipopolysaccharide-induced TNF-α, with less efficiency than propionate or butyrate (71, 72). However, propionate provides resistance against the expansion of pathogenic bacteria, such as *Salmonella* and *Shigella*, by disrupting the balance of their intracellular pH (73).

Furthermore, isobutyrate can be used as an alternative energy source by the intestinal epithelial cells when the butyrate is scarce (74), which is frequently the case under *Salmonella* infection in chicken broilers. The valerate role is still unclear, but it’s known as a potential inhibitor of cancerous cells’ growth (75). The SCFAs previously cited were significantly higher in low H/L ratio chickens than in high H/L ratio chickens. These results suggest that these SCFAs contribute to the disease resistance observed with low H/L ratio chickens under *Salmonella* infection.

In this study, *Firmicutes*, *Bacteroidetes*, and *Proteobacteria* were the more abundant phyla in the cecal microbiota of high and low H/L ratio SE-infected chickens, as previously reported (76–78). Increasing evidence emphasized the role of some bacteria phyla such as *Firmicutes*, *Bacteroidetes*, *Proteobacteria*, *Actinobacteria*, and others, in defense of the host against pathogenic microorganisms (79, 80). Nevertheless, it was noteworthy that low and high H/L ratio chickens exhibited significantly different cecal microbiota composition over time points post-infection. We observed that under SE infection, low H/L ratio chickens displayed significantly increased abundance in *Proteobacteria* and *Bacteroidetes* at 7 and 21 days after infection, respectively. Although, the *Proteobacteria* phylum includes many pathogenic bacteria, such as *Escherichia* Coli, *Helicobacter*, *Vibrio* Cholera, and *Salmonella* spp. A recent study demonstrated the role of bacteria from the *Enterobacteriaceae* family, particularly *Escherichia* Coli, in protecting the chicken against *Salmonella* colonization and enhance immune responses (81–83). Litvak et colleagues (83) established that spore-forming bacteria and commensal *Enterobacteriaceae* confer resistance on chickens against *Salmonella* colonization. They discovered that *Salmonella* uses its virulence factors to boost oxygen levels in the intestinal epithelium, whereas *Escherichia* Coli, a member of the *Enterobacteriaceae*, confers colonization resistance on *Salmonella* by competing for oxygen with it. In neonatal mice, *Clostridia* and *Proteobacteria* contribute to the niche protection through early colonization of *Proteobacteria* (84, 85).

*Bacteroidetes* are gram-negative bacteria, including strict aerobes and anaerobes (86). Among the genera of *Bacteroidetes* phylum, *Bacteroides* was the more abundant genus in this study. *Bacteroides* species possess a cluster of genes coding to produce polysaccharides (87, 88). Among those polysaccharides, polysaccharide A (PSA) confers the capacity of gastrointestinal tract colonization, improves survival in the gut, and stimulates the host immune system (89, 90). Wrzosek and colleagues (91) demonstrated that *Bacteroides thetaiotaomicron*, a producer of acetate, induces goblet cell differentiation, increasing mucin gene expression and goblet cell number. Consistent with our findings, we observed that low H/L ratio chickens exhibited a significant abundance of *Bacteroidetes* at 21 dpi.

Moreover, we also observed that the low H/L ratio chickens displayed significantly increased goblet cell numbers in the ileal villi and crypts at 7 and 21 dpi. Interestingly, our results revealed that low H/L ratio chickens showed significantly higher acetate cecal content at 7 and 21 dpi. However, in this study, the *Bacteroides* species identified was *Bacteroides plebeius*. Zhu et collaborators (92) demonstrated that 1,25-dihydroxy vitamin D3 (1,25(OH)2D3) deficiency impairs the production of metabolites and the composition of the gut microbiota. The same deficit impaired *Bacteroides Plebeius* and SCFAs production, associated with a thinner mucus layer and increasing bacterial translocation to the mesenteric lymph nodes. The mucus layers protect against the invasion of microorganisms and pathogens in diverse ways. These results suggest that *Proteobacteria* and *Bacteroides* could contribute to the resistance to *Salmonella* Enteritidis observed in low H/L ratio chickens through oxygen competition and mucins production enhancement, respectively. These hypotheses leave us to suppose that this process can be one of the intestinal microbiota’s immunomodulatory mechanisms, conferring host resistance to *Salmonella* infection in chicken broilers.

From the correlation analysis between the cecal microbiota genus and the environmental factors such as the H/L ratio and the SCFAs content, we observed that *Anaerostipes*, *Lachnoclostridium*, and unclassified CHKCI002 genus had a significant positive relationship with the H/L ratio. Moreover, *Bacteroides* had a significant positive correlation with body weight post-infection, propionate and valerate contents. At the same time, we found that *Salmonella* spp. and *Escherichia shigella* had a significant negative correlation with body weight post-infection, propionate and valerate contents. *Anaerostipes* are Gram-variable bacteria, initially, they are Gram-positive, but in old culture (> 16 hours), they are Gram-negative. *Anaerostipes* bacteria are simultaneously acetate users and butyrate producers (93). The butyrate produced by commensal microorganisms plays an essential role in adjusting host reactions to inflammatory lesions (94). However, Deaver and colleagues (95) reported *Ruminococcus torques* are potential bacterial species causing a decrease in gut integrity. In accordance with this previous study (95), the current study found an increased abundance of *Ruminococcus torques* in high H/L ratio chickens (7dpi), which, compared to low H/L ratio chickens, exhibited significantly reduced goblet cells number. In mice subjected to abnormal light exposition, the increase in *Ruminococcus torques* and decreased *Lactobacillus johnsonii* were associated with a reduction in intestinal health and immune barrier function (95). Previous studies showed that *Ruminococcus torques* are a potential mucus degrader, which may disrupt gut barrier integrity (96, 97). The decrease in goblet cells observed seven days after SE infection in high H/L ratio chickens can be attributed to these bacteria. These results suggest that low H/L ratio chickens display a favorable gut microbiota for the preservation of intestinal health and gut mucosa integrity under *Salmonella* infection in chicken broilers. Moreover, the species *Bacteroides* plebeius could be involved in this improved resistance against *Salmonella* infection observed with low H/L ratio chickens.

This study identified the antibiotics resistance genes (ARGs) and the virulence factors (VFs) from the cecal microbiota of high and low H/L ratio SE-infected chickens. In addition, the contributions of the bacterial genus and the distributions among high and low H/L ratio chickens over time points post-infection were also determined. Following the previous works (98, 99), the genera bacteria belonging to *Firmicutes, Bacteroidetes* and *Proteobacteria* contributed more abundantly to the ARGs. Moreover, our results revealed that the low H/L ratio chickens exhibited fewer ARGs and VFs than the high H/L ratio chickens under SE infection. These results suggest that low H/L ratio chickens can be more suitable for the selection of disease-resistant chickens in breeding programs.

Previous studies demonstrated that chickens and others avian species with low H/L ratio are more resistant to environmental stressors than birds with high H/L ratio (23, 24). Recent researches even reported that a low H/L confers resistance advantages such as enhanced immune response, performances, adaptability, long life span and survival (25, 48). In the present study, we hypothesized that the H/L ratio is associated with the microbiome composition, which provides the differential resistance observed between individuals with high and low H/L ratios. Our results showed that under *Salmonella* infection, chickens with low and high H/L ratio display different cecal microbiome compositions and that the chickens with low H/L ratio display beneficial microbiome composition than chickens with high H/L. These results could lead us to conclude that the H/L ratio modulates the microbiome composition, which influences the resistance to *Salmonella* infection. Unfortunately, an obstacle to the generalization was the absence of a control group before infection and 21 days after infection. Therefore, some questions remain unelucidated, what is the changes with the time of gut microbiome in chickens displaying differential H/L ratio? Is the microbiome composition responsible for the differential resistance observed between individuals with high and low H/L?

As with the majority of studies, the current study’s design is subject to some limitations that could be addressed in future research. The primary limitation to the generalization of these results is the absence of sampling before infection and the control group at 21 dpi. The second limitation concerns the sample size. It is the case of histomorphology results, villi morphometry and goblet cells number, for which we used only two biological replicates. Such a small sample size limits our study’s generalization to a potential association of the H/L ratio with the intestinal barrier. It is possible to suggest that the H/L ratio is associated with intestinal barrier and microbiome composition, but the sample size used in the present work constitutes an important limitation to lead us to this conclusion. Future studies should consider these limitations to effectively expand the knowledge related to the complex interactions among chicken, Salmonella, and Gut-microbiome.

## Conclusion

The conclusion emerging from this study is that chickens with a low H/L ratio are more resistant to *Salmonella* Enteritidis infection. The microbiome diversity and functional capacity exploration revealed that chickens with high and low H/L ratios display significantly different microbiota composition at 21 dpi, with beneficial bacterial taxa identified in low H/L ratio chickens, compared to high H/L ratio chickens under *Salmonella* infection. From the results generated in this study, it is possible to suggest that the commensal *Proteobacteria* and *Bacteroidetes* are involved in this resistance against *Salmonella* through diverse mechanisms not well understood. The present study has provided new evidence of using the H/L ratio as an immune index in chicken broilers and contributed to a better understanding of the role and mechanisms of gut microbiota in shaping the host immune system against *Salmonella* infection.

## Data Availability Statement

The datasets analyzed for this study can be found in the Genome Sequence Archive in BIG Data Center (100), Beijing Institute of Genomics (BIG), Chinese Academy of Sciences, under project number PRJCA008896 and accession number CRA006497 that are publicly accessible at http://bigd.big.ac.cn/gsa.

## Ethics Statement

The animal study was reviewed and approved by Institute of Animal Sciences’ Animal Welfare Committee (Chinese Academy of Agricultural Sciences, Beijing, China).

## Author Contributions

MT and QW contributed to the formulation and design of the experiment, performed the study, analyzed the data, and drafted the manuscript. ALBS, JZ, and JWa contributed to the sample collection and laboratory analysis. MT, QW, ALBS, JZ, JWa, JD, HW, QW, and NZ contributed to the investigation and the sample collection. QL contributed to the suggestion of data analysis and interpretation. JWe and GZ contributed to the conception and design of the study and modified the manuscript. MT and QW revised and edited the manuscript. All authors contributed to the article and approved the submitted version.

## Funding

This research was supported by grants from the National Natural Science Foundation of China (No. 32102533), National Key R&D Program of China (2018YFE0128000), China Agriculture Research System of MOF and MARA (CARS-41), and the Agricultural Science and Technology Innovation Program (ASTIP-IAS04 and CAASZDRW202005).

## Conflict of Interest

The authors declare that the research was conducted in the absence of any commercial or financial relationships that could be construed as a potential conflict of interest.

## Publisher’s Note

All claims expressed in this article are solely those of the authors and do not necessarily represent those of their affiliated organizations, or those of the publisher, the editors and the reviewers. Any product that may be evaluated in this article, or claim that may be made by its manufacturer, is not guaranteed or endorsed by the publisher.

## Supplementary Material

Supplementary Figure 1 Visual representation of the experimental design.

Supplementary Figure 2 Venn diagram ASVs **(A)** and genus **(B)** levels. H7_CN: high H/L non-infected 7 dpi (n = 5); L7_CN: low H/L non-infected 7 dpi (n = 5); H7_SE: High H/L SE-infected 7 dpi (n = 8); L7_SE: Low H/L SE-infected 7 dpi (n = 7); H21_SE: high H/L SE-infected 21 dpi (n = 6); L21_SE: Low H/L SE-infected 21 dpi (n = 7).

Supplementary Figure 3 Comparative analysis of relative abundance of cecal microbiota phyla and genera. **(A)** Cecal microbiota phyla differential abundance between non-infected and SE-infected high H/L ratio chickens at 7 dpi. **(B)** Cecal microbiota genera differential abundance between non-infected and SE-infected high H/L ratio chickens at 7 dpi. **(C)** Cecal microbiota phyla differential abundance between non-infected and SE-infected low H/L ratio chickens at 7 dpi. **(D)** Cecal microbiota phyla differential abundance between non-infected and SE-infected low H/L ratio chickens at 7 dpi. Data analyzed by Wilcoxon rank-sum test, with reported p-value and significances. H7_CN: high H/L non-infected 7 dpi (n = 5); L7_CN: low H/L non-infected 7 dpi (n = 5); H7_SE: High H/L SE-infected 7 dpi (n = 8); L7_SE: Low H/L SE-infected 7 dpi (n = 7).

Supplementary Figure 4 Cecal microbiome composition based on the metagenomic sequencing. **(A)** four groups top differentially abundant bacterial phyla; **(B)** four groups top differentially abundant bacterial genera; **(C)** four groups top differentially abundant bacterial species; H7: High H/L SE-infected 7 dpi (n = 7); L7: Low H/L SE-infected 7 dpi (n = 6); H21: high H/L SE-infected 21 dpi (n = 6); L21: Low H/L SE-infected 21 dpi (n = 7).

Supplementary Figure 5 Gene and functional capacity relative abundance. **(A)** Venn diagram showing the number of shared and unique genes between the four groups based on the non-redundant database; **(B)** Group-wise KEEG pathways distribution bar plot. H7: High H/L SE-infected 7 dpi (n = 7); L7: Low H/L SE-infected 7 dpi (n = 6); H21: high H/L SE-infected 21 dpi (n = 6); L21: Low H/L SE-infected 21 dpi (n = 7).

Supplementary Figure 6 Gene and functional capacity relative abundance (Circos analysis). **(A)** Comparative relative abundance of CARD functional terms among the four groups of microbiomes; **(B)** Comparative relative abundance of VFs functional terms among the four groups of microbiomes. H7: High H/L SE-infected 7 dpi (n = 7); L7: Low H/L SE-infected 7 dpi (n = 6); H21: high H/L SE-infected 21 dpi (n = 6); L21: Low H/L SE-infected 21 dpi (n = 7).

Supplementary Figure 7 LEfSe of KEGG pathways level 3 between low and high H/L ratio SE-infected chicken cecal microbiota at 7 and 21 dpi. H7: High H/L SE-infected 7 dpi (n = 7); L7: Low H/L SE-infected 7 dpi (n = 6); H21: high H/L SE-infected 21 dpi (n = 6); L21: Low H/L SE-infected 21 dpi (n = 7).

Supplementary Figure 8 Antibiotic Resistance Genes (ARGs) types and genes based CARD databases, and microbiota species contribution. **(A, B)** ARGs differentially abundant between high and low H/L chickens infected by *Salmonella* at 7 and 21 dpi, based CARD database. **(C)** cecal genera and ARO contribution. H7: High H/L SE-infected 7 dpi (n = 7); L7: Low H/L SE-infected 7 dpi (n = 6); H21: high H/L SE-infected 21 dpi (n = 6); L21: Low H/L SE-infected 21 dpi (n = 7).

Supplementary Figure 9 Virulence factors and microbiota species contribution. **(A, B)** virulence factors differentially abundant between high and low H/L ratio chickens infected by *Salmonella* at 7 and 21 dpi. **(C)** cecal microbiota genera and virulence factors contribution at the level 2. **(D)** cecal microbiota genera and virulence factors contribution. H7: High H/L SE-infected 7 dpi (n = 7); L7: Low H/L SE-infected 7 dpi (n = 6); H21: high H/L SE-infected 21 dpi (n = 6); L21: Low H/L SE-infected 21 dpi (n = 7).
