# Supplementary figures and images for: Heterophil/Lymphocyte Ratio Level Modulates Salmonella Resistance, Cecal Microbiota Composition and Functional Capacity in Infected Chicken
Source: Front Immunol. 2022 Apr 14;13:816689. doi: 10.3389/fimmu.2022.816689 (PMC9047862; doi:10.3389/fimmu.2022.816689)

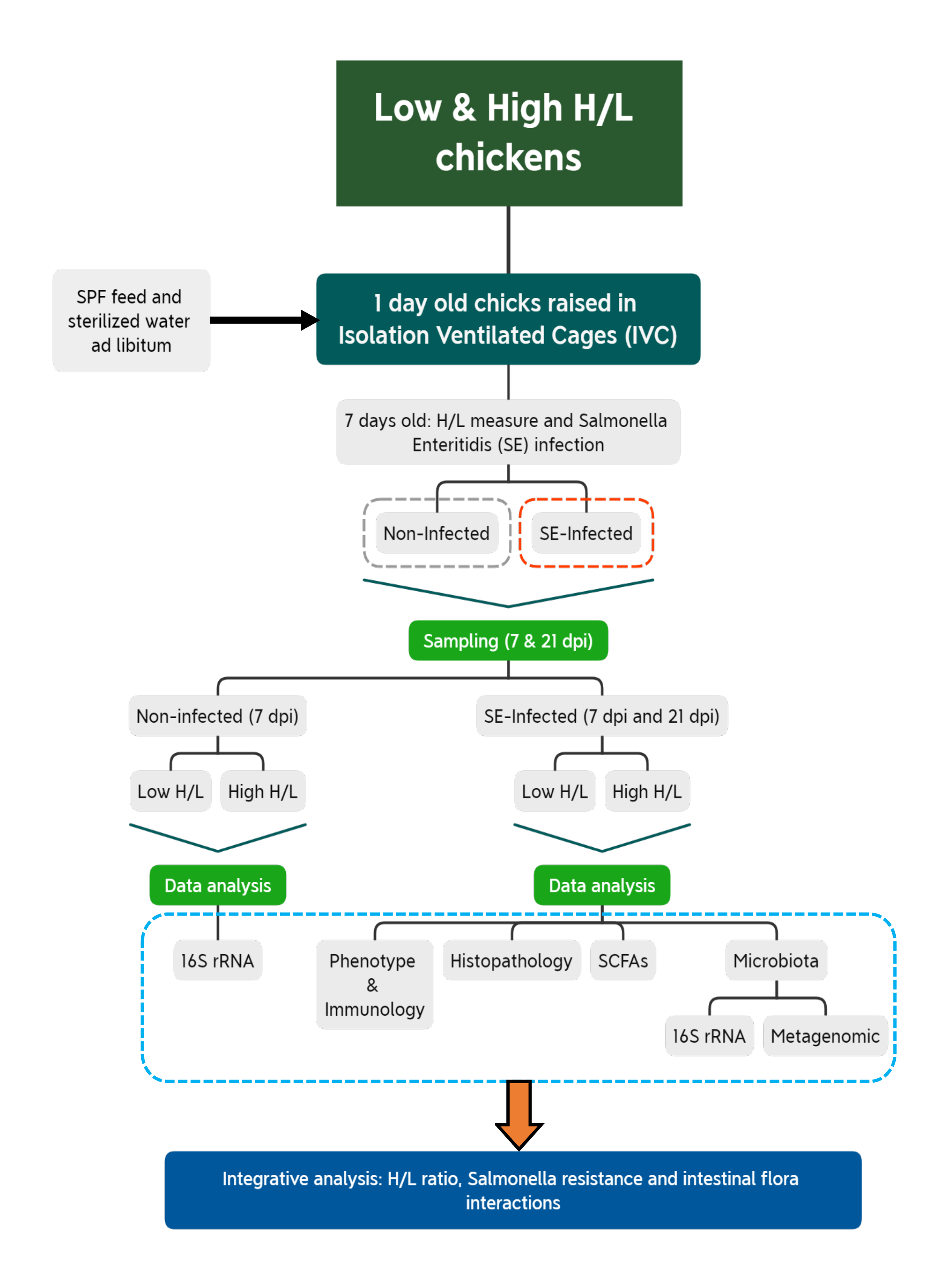

Supplement: Supplementary Figure 1 — Visual representation of the experimental design. [file Image_1.tiff]

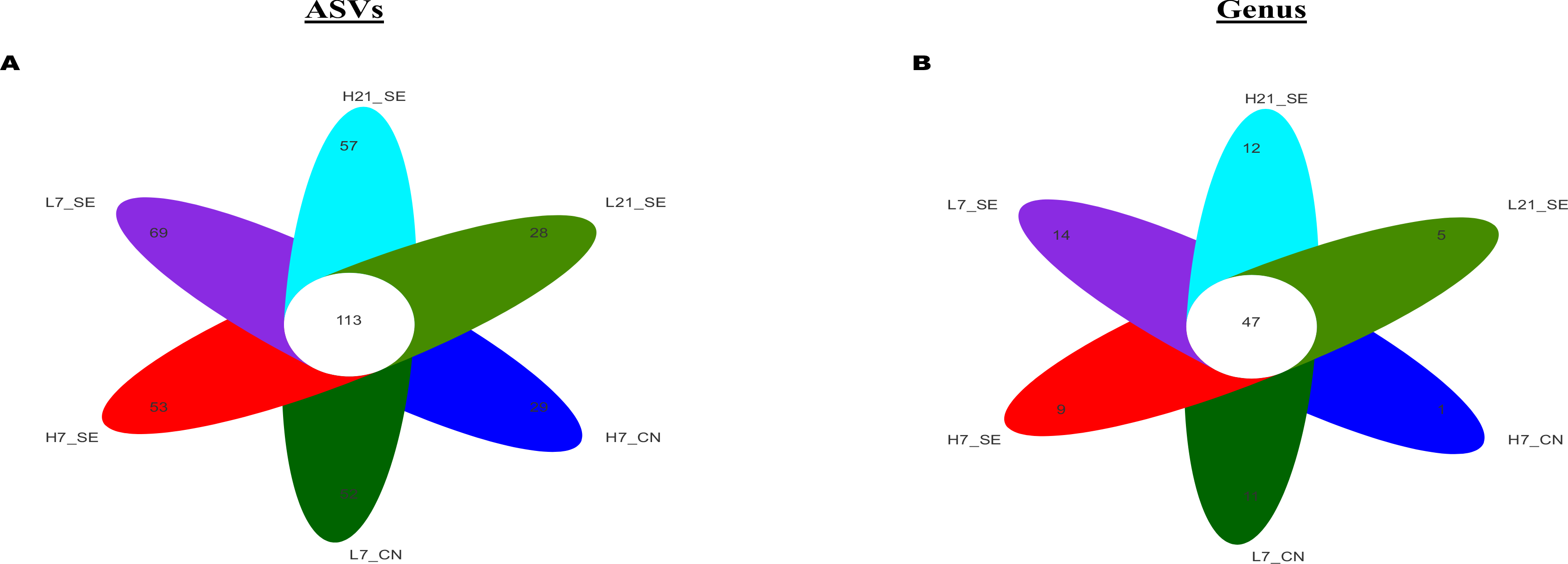

Supplement: Supplementary Figure 2 — Venn diagram ASVs (A) and genus (B) levels. H7_CN: high H/L non-infected 7 dpi (n = 5); L7_CN: low H/L non-infected 7 dpi (n = 5); H7_SE: High H/L SE-infected 7 dpi (n = 8); L7_SE: Low H/L SE-infected 7 dpi (n = 7); H21_SE: high H/L SE-infected 21 dpi (n = 6); L21_SE: Low H/L SE-infected 21 dpi (n = 7). [file Image_2.tiff]

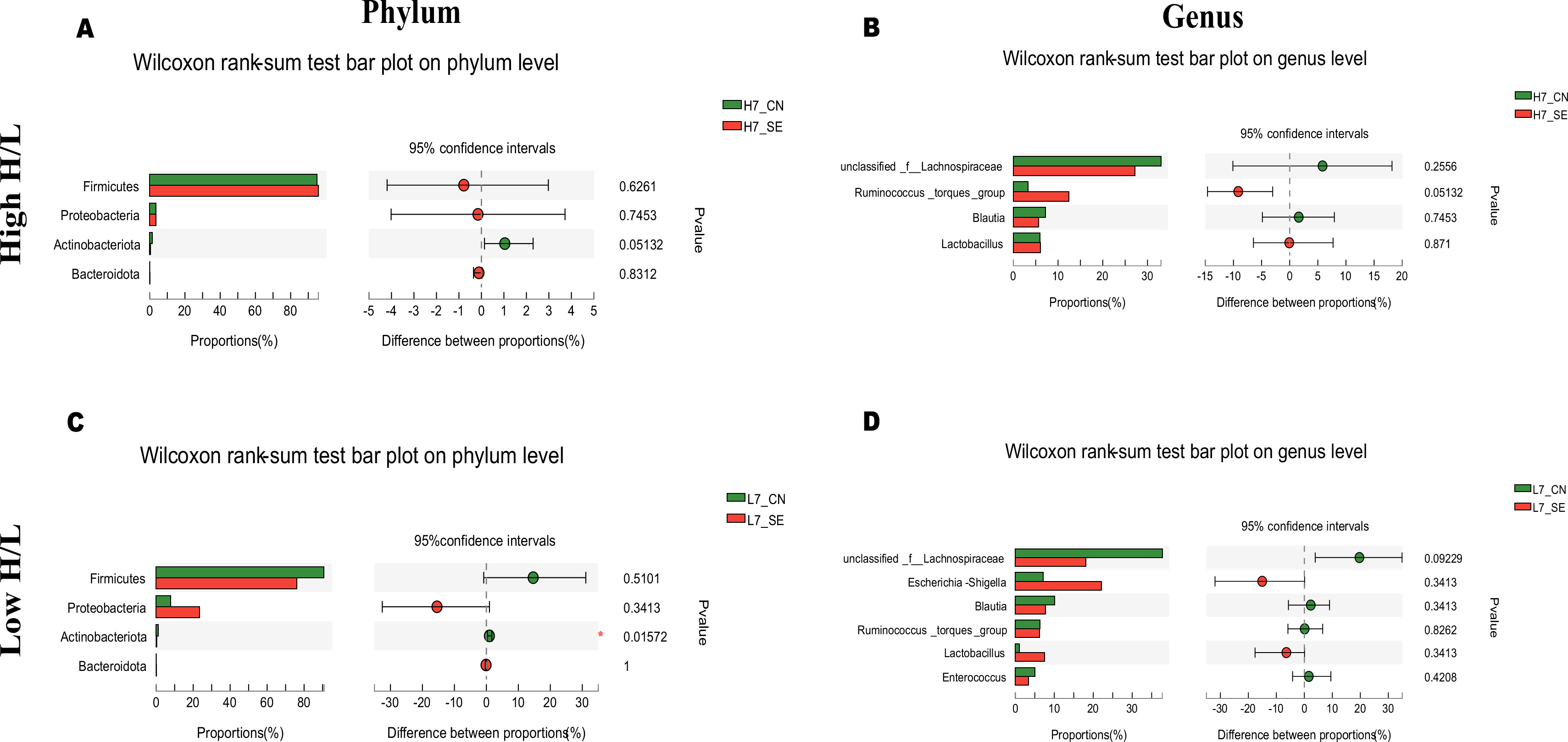

Supplement: Supplementary Figure 3 — Comparative analysis of relative abundance of cecal microbiota phyla and genera. (A) Cecal microbiota phyla differential abundance between non-infected and SE-infected high H/L ratio chickens at 7 dpi. (B) Cecal microbiota genera differential abundance between non-infected and SE-infected high H/L ratio chickens at 7 dpi. (C) Cecal microbiota phyla differential abundance between non-infected and SE-infected low H/L ratio chickens at 7 dpi. (D) Cecal microbiota phyla differential abundance between non-infected and SE-infected low H/L ratio chickens at 7 dpi. Data analyzed by Wilcoxon rank-sum test, with reported p-value and significances. H7_CN: high H/L non-infected 7 dpi (n = 5); L7_CN: low H/L non-infected 7 dpi (n = 5); H7_SE: High H/L SE-infected 7 dpi (n = 8); L7_SE: Low H/L SE-infected 7 dpi (n = 7). [file Image_3.tiff]

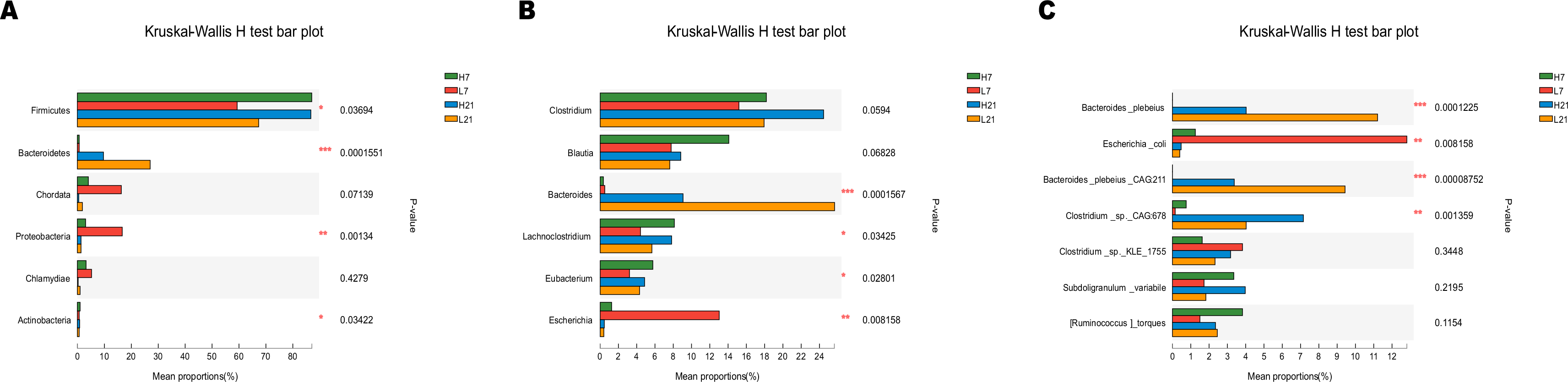

Supplement: Supplementary Figure 4 — Cecal microbiome composition based on the metagenomic sequencing. (A) four groups top differentially abundant bacterial phyla; (B) four groups top differentially abundant bacterial genera; (C) four groups top differentially abundant bacterial species; H7: High H/L SE-infected 7 dpi (n = 7); L7: Low H/L SE-infected 7 dpi (n = 6); H21: high H/L SE-infected 21 dpi (n = 6); L21: Low H/L SE-infected 21 dpi (n = 7). [file Image_4.tiff]

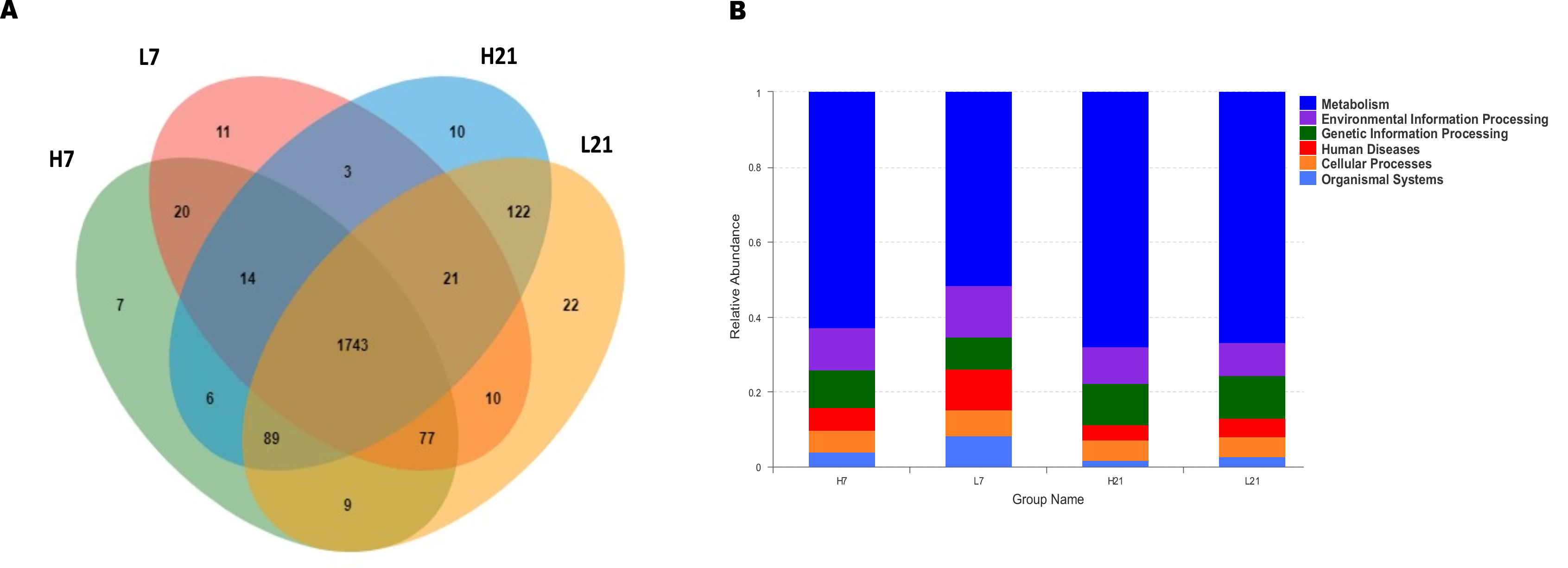

Supplement: Supplementary Figure 5 — Gene and functional capacity relative abundance. (A) Venn diagram showing the number of shared and unique genes between the four groups based on the non-redundant database; (B) Group-wise KEEG pathways distribution bar plot. H7: High H/L SE-infected 7 dpi (n = 7); L7: Low H/L SE-infected 7 dpi (n = 6); H21: high H/L SE-infected 21 dpi (n = 6); L21: Low H/L SE-infected 21 dpi (n = 7). [file Image_5.tiff]

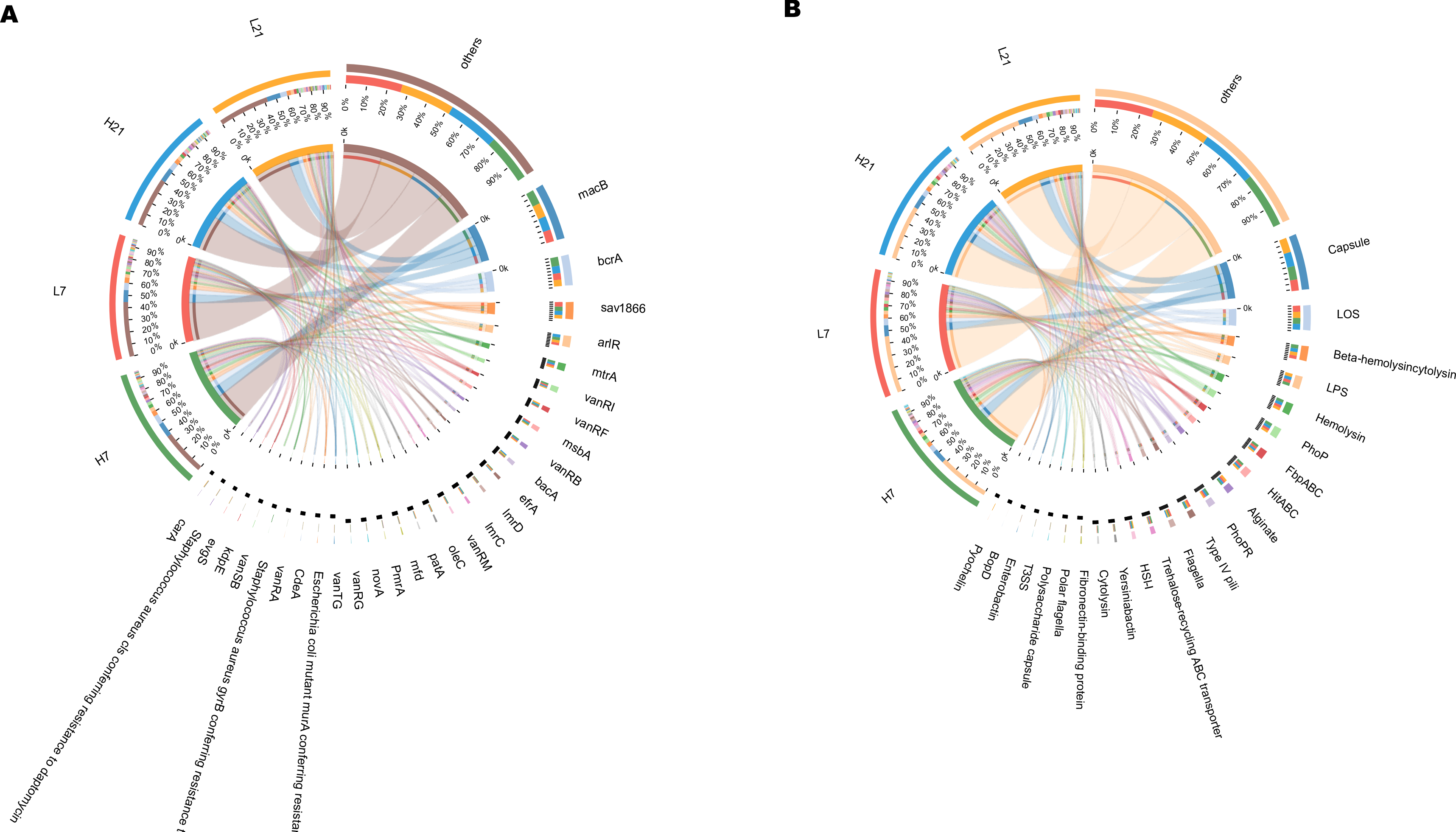

Supplement: Supplementary Figure 6 — Gene and functional capacity relative abundance (Circos analysis). (A) Comparative relative abundance of CARD functional terms among the four groups of microbiomes; (B) Comparative relative abundance of VFs functional terms among the four groups of microbiomes. H7: High H/L SE-infected 7 dpi (n = 7); L7: Low H/L SE-infected 7 dpi (n = 6); H21: high H/L SE-infected 21 dpi (n = 6); L21: Low H/L SE-infected 21 dpi (n = 7). [file Image_6.tiff]

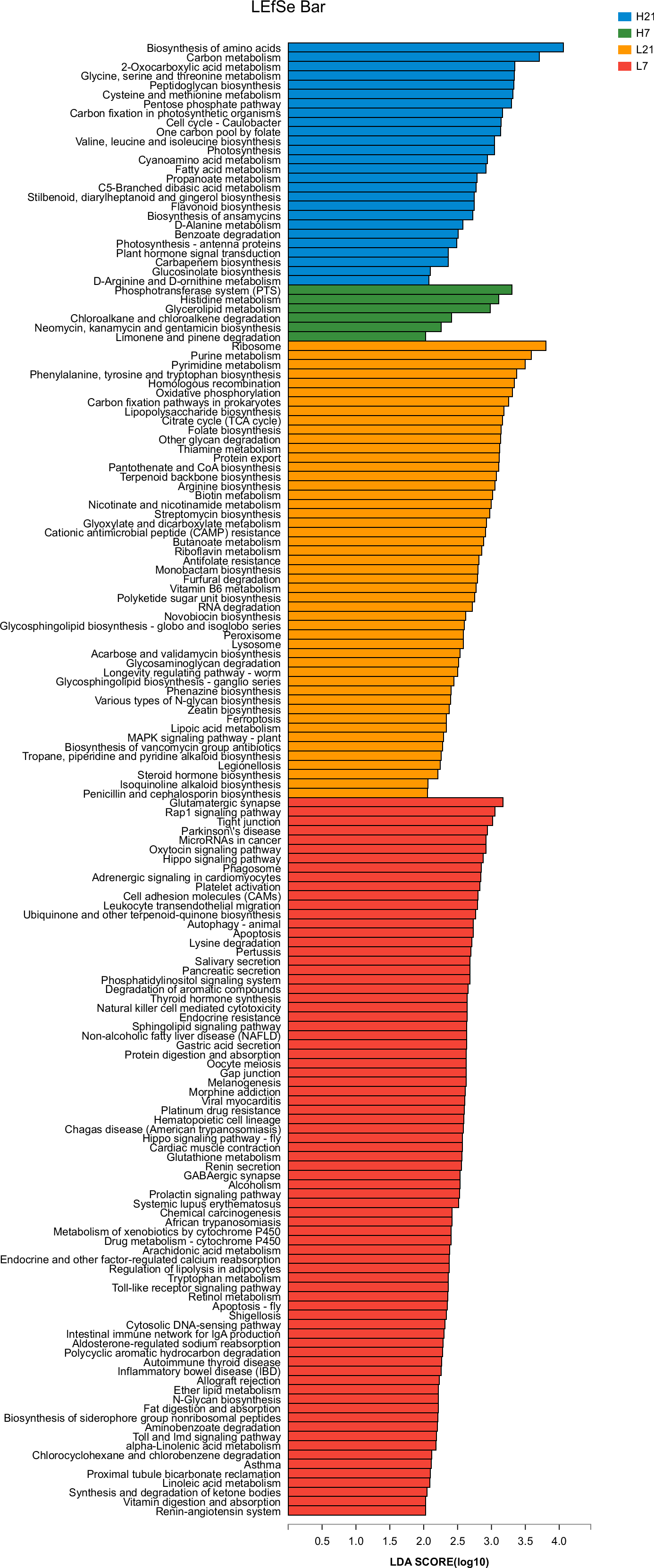

Supplement: Supplementary Figure 7 — LEfSe of KEGG pathways level 3 between low and high H/L ratio SE-infected chicken cecal microbiota at 7 and 21 dpi. H7: High H/L SE-infected 7 dpi (n = 7); L7: Low H/L SE-infected 7 dpi (n = 6); H21: high H/L SE-infected 21 dpi (n = 6); L21: Low H/L SE-infected 21 dpi (n = 7). [file Image_7.tiff]

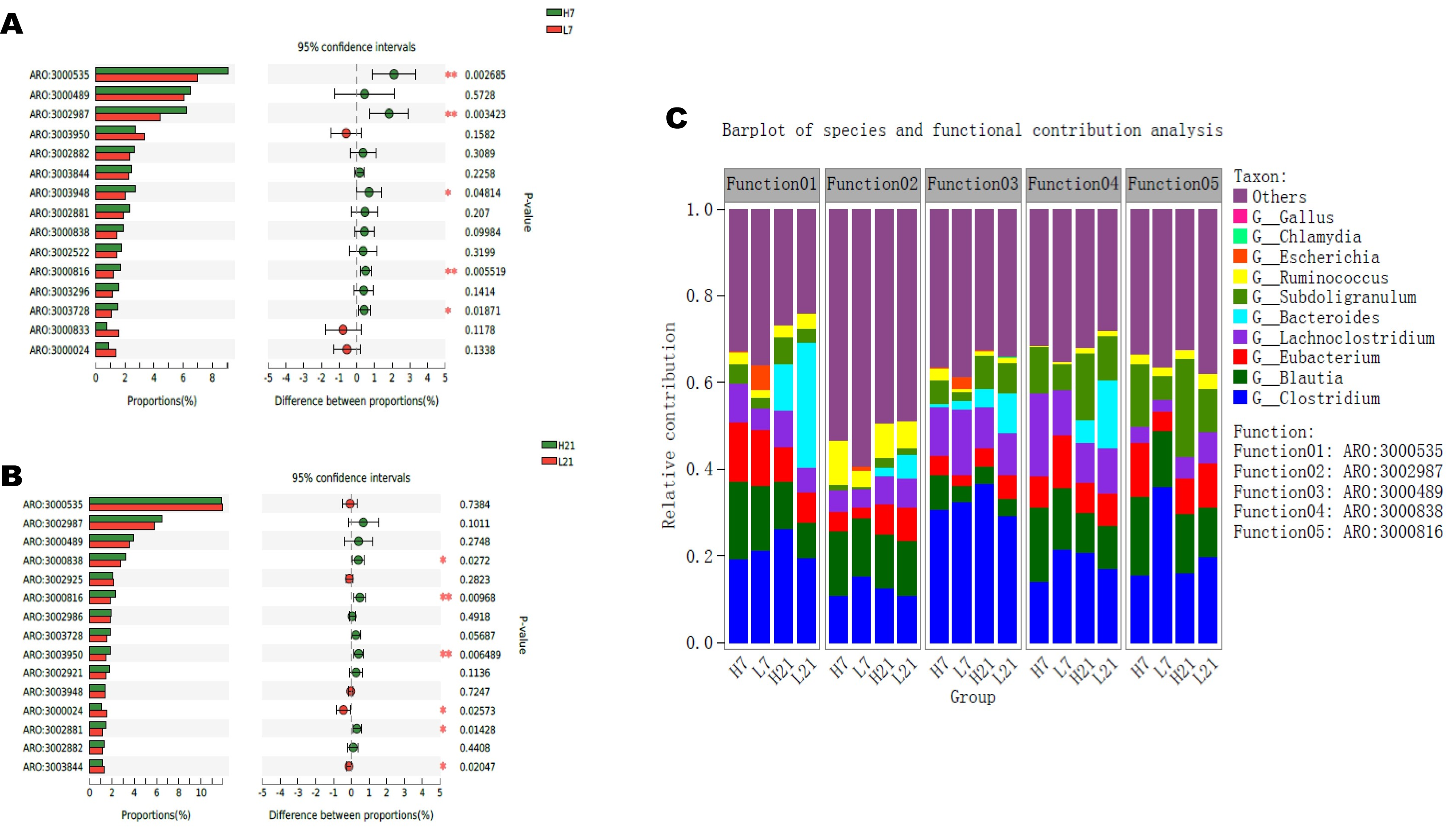

Supplement: Supplementary Figure 8 — Antibiotic Resistance Genes (ARGs) types and genes based CARD databases, and microbiota species contribution. (A, B) ARGs differentially abundant between high and low H/L chickens infected by Salmonella at 7 and 21 dpi, based CARD database. (C) cecal genera and ARO contribution. H7: High H/L SE-infected 7 dpi (n = 7); L7: Low H/L SE-infected 7 dpi (n = 6); H21: high H/L SE-infected 21 dpi (n = 6); L21: Low H/L SE-infected 21 dpi (n = 7). [file Image_8.tiff]

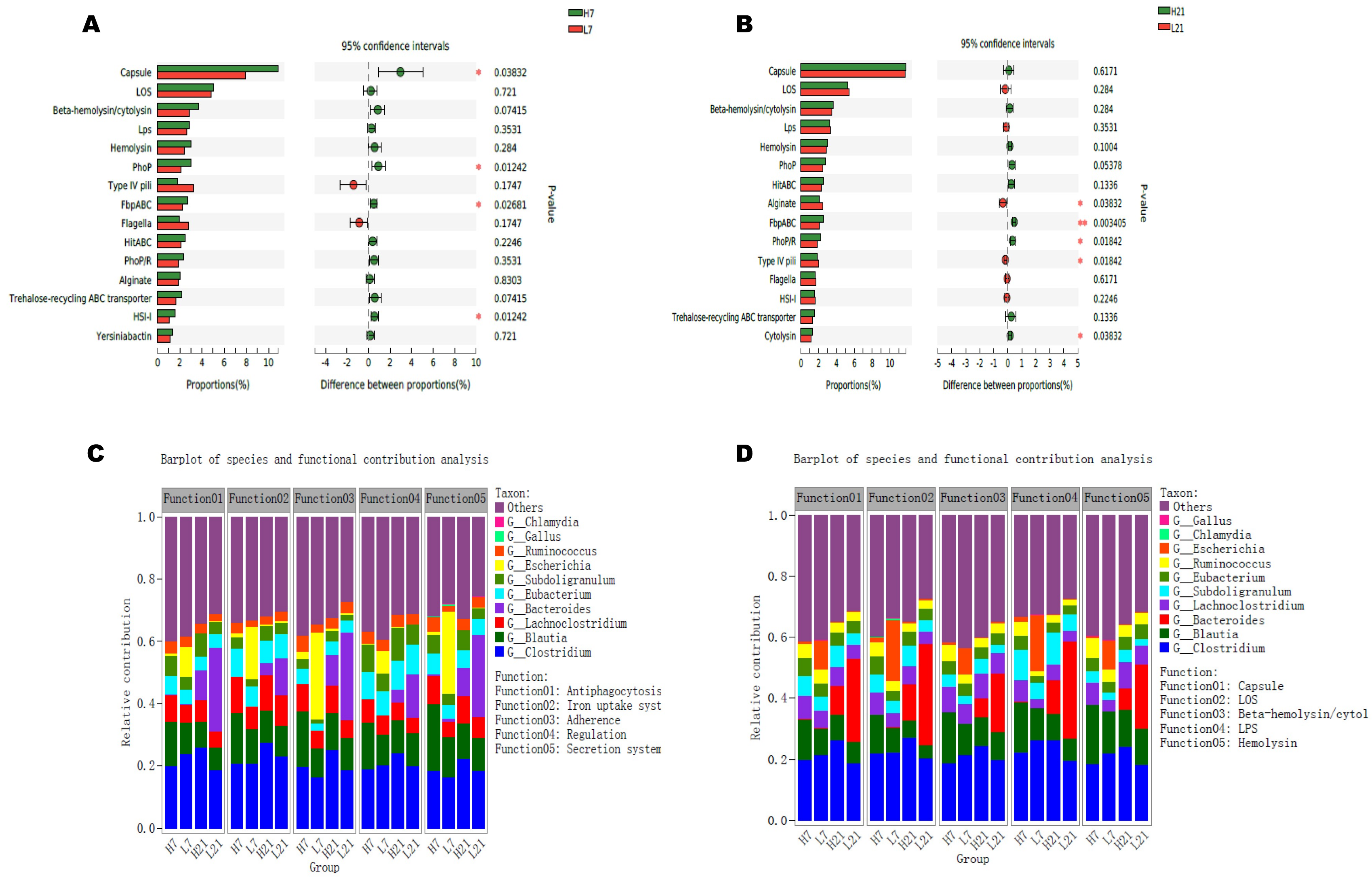

Supplement: Supplementary Figure 9 — Virulence factors and microbiota species contribution. (A, B) virulence factors differentially abundant between high and low H/L ratio chickens infected by Salmonella at 7 and 21 dpi. (C) cecal microbiota genera and virulence factors contribution at the level 2. (D) cecal microbiota genera and virulence factors contribution. H7: High H/L SE-infected 7 dpi (n = 7); L7: Low H/L SE-infected 7 dpi (n = 6); H21: high H/L SE-infected 21 dpi (n = 6); L21: Low H/L SE-infected 21 dpi (n = 7). [file Image_9.tiff]
